# From neuraminidase inhibitors to novel mechanisms: expanding therapeutic strategies against influenza

**DOI:** 10.3389/fcimb.2026.1859541

**Published:** 2026-07-24

**Authors:** Yan Sun, Puongtip Kunanusorn, Kuang Ming Tup, Santosh Chokkakula, Bing Yang

**Affiliations:** 1 Department of Public Health, International School, Krirk University, Bangkok, Thailand; 2 Department of Medical and Nursing Science, International College, Krirk University, Bangkok, Thailand; 3 Department of Microbiology, Chungbuk National University College of Medicine and Medical Research Institute, Cheongju, Republic of Korea; 4 Department of Cell Biology, College of Basic Medical Sciences, Tianjin Medical University, Tianjin, China

**Keywords:** antiviral resistance, antiviral therapy, cap-dependent endonuclease inhibitor, H5N1, host-directed therapy, influenza virus, neuraminidase inhibitors, novel antiviral agents

## Abstract

Seasonal influenza causes approximately one billion infections and up to 650,000 respiratory deaths annually. Despite decades of antiviral development, the therapeutic arsenal remains constrained by time-dependent efficacy (clinical benefit confined largely to treatment initiated within 48 hours of symptom onset), low genetic resistance barriers (resistance arising from a single point mutation), and critical evidence gaps in high-risk populations. This review critically evaluates the antiviral landscape, focusing on next-generation cap-dependent endonuclease inhibitors (CENIs), resistance implications across all approved drug classes, translational barriers in host-directed therapies and biologics, and H5N1 pandemic preparedness. Suraxavir marboxil, ZX-7101A, and the first approved polymerase basic protein 2 (PB2) inhibitor, onradivir, confirm that scaffold-level optimization (incremental structural refinement within an existing drug class) can meaningfully reduce resistance emergence rates. Across all approved classes, a shared failure pattern is evident: single-target dependence, low-fitness-cost resistance mutations (mutations that confer drug resistance without measurably impairing viral replication or transmission), and a therapeutic window that most patients do not reach. Combination antiviral therapy shows promise for resistance prevention, though the FLAGSTONE Phase 3 trial demonstrated that virological benefit does not consistently translate to clinical outcomes in hospitalized patients. Host-directed therapies and biologics face a shared translational obstacle: the biological window of maximal activity consistently precedes the clinical window of patient presentation, and systemic biologics cannot achieve inhibitory concentrations at the respiratory mucosal surface. For H5N1, preparedness relies on a single observationally supported drug class; baloxavir is absent from most national stockpiles, and no randomized trial data exists for any antiviral in human H5N1 infection. Adaptive trial infrastructure, diversified stockpiling to include next-generation CENIs, and equitable global antiviral access are the most urgent priorities in influenza therapeutics.

## 1 Introduction

Influenza viruses are among the most significant respiratory pathogens globally and are responsible for both seasonal epidemics and pandemics. Seasonal influenza affects around 1 billion individuals each year, mainly during the winter months. According to the World Health Organization (WHO), 3 to 5 million cases of severe illness arise each year, with 290,000 to 650,000 respiratory deaths (The Burden of Influenza; Iuliano et al., 2018). The 2024–2025 influenza season was one of the worst in recent years, characterized by co-circulation of influenza A(H3N2), A(H1N1)pdm09, and B/Victoria lineage viruses, with A (H3N2) predominating in several WHO regions (Seasonal Influenza - Global Situation). Preliminary surveillance data for the 2025–2026 season have identified the emergence of a novel A(H3N2) subclade K (formerly J.2.4.1), carrying multiple key hemagglutinin substitutions relative to currently deployed vaccine strains, raising early concerns regarding potential antigenic mismatch and underscoring the continuing limitations of strain-matched vaccine strategies (Dapat et al., 2025; Kirsebom et al., 2025; Zambon and Hayden, 2026). Preliminary in-season predictions for the 2025–2026 US flu season indicate a significant disease burden. As of early 2026, the Centers for Disease Control and Prevention (CDC) estimates around 15 million flu cases, 180,000 hospital admissions, and 7,400 fatalities from influenza, with end-of-season finalized figures not yet available at the time of writing (CDC, 2025). Influenza viruses have pandemic potential due to their segmented genomic structure, which allows for reassortment, interspecies transmission, and the ability to elude population protection through antigenic drift and shift (Neumann et al., 2025). Additionally, concerns about highly pathogenic avian influenza viruses like H5N1 and H7N9 highlight the constant threat posed by these viruses (El-Bidawy et al., 2025).

Annual vaccination remains the primary prevention strategy, but its effectiveness fluctuates between 10% and 60% depending on the season (Belongia et al., 2016). Key limitations include antigenic mismatch between vaccine and circulating strains, egg-based production timelines of 6–8 months, and impaired vaccine responses in the elderly and immunocompromised (Seasonal Influenza - Global Situation; CDC, 2025).

In the context of these vaccine limitations, antiviral therapies serve a complementary and critical role by providing direct treatment and control once infection has started. Neuraminidase inhibitors such as oseltamivir have been shown in randomized controlled studies to considerably shorten the duration of symptoms and viral shedding, when started within 48 hours of the onset of symptoms (Nicholson et al., 2000). Antiviral drugs are useful for post-exposure prophylaxis besides treating established infections. This reduces the prevalence of laboratory-confirmed influenza, especially in high-risk patients and household contacts (Welliver et al., 2001). Antivirals are also essential for pandemic preparedness and outbreak control in institutional settings, particularly in the early phases of viral emergence when vaccine availability is limited. According to the observational data from the 2009 H1N1 pandemic, antiviral therapy was linked to lower mortality in hospitalized patients. The greatest benefit was seen when therapy was started early, but the benefit was still noticeable when it was started later in the disease course (Rodríguez et al., 2011).

**Figure 1:**
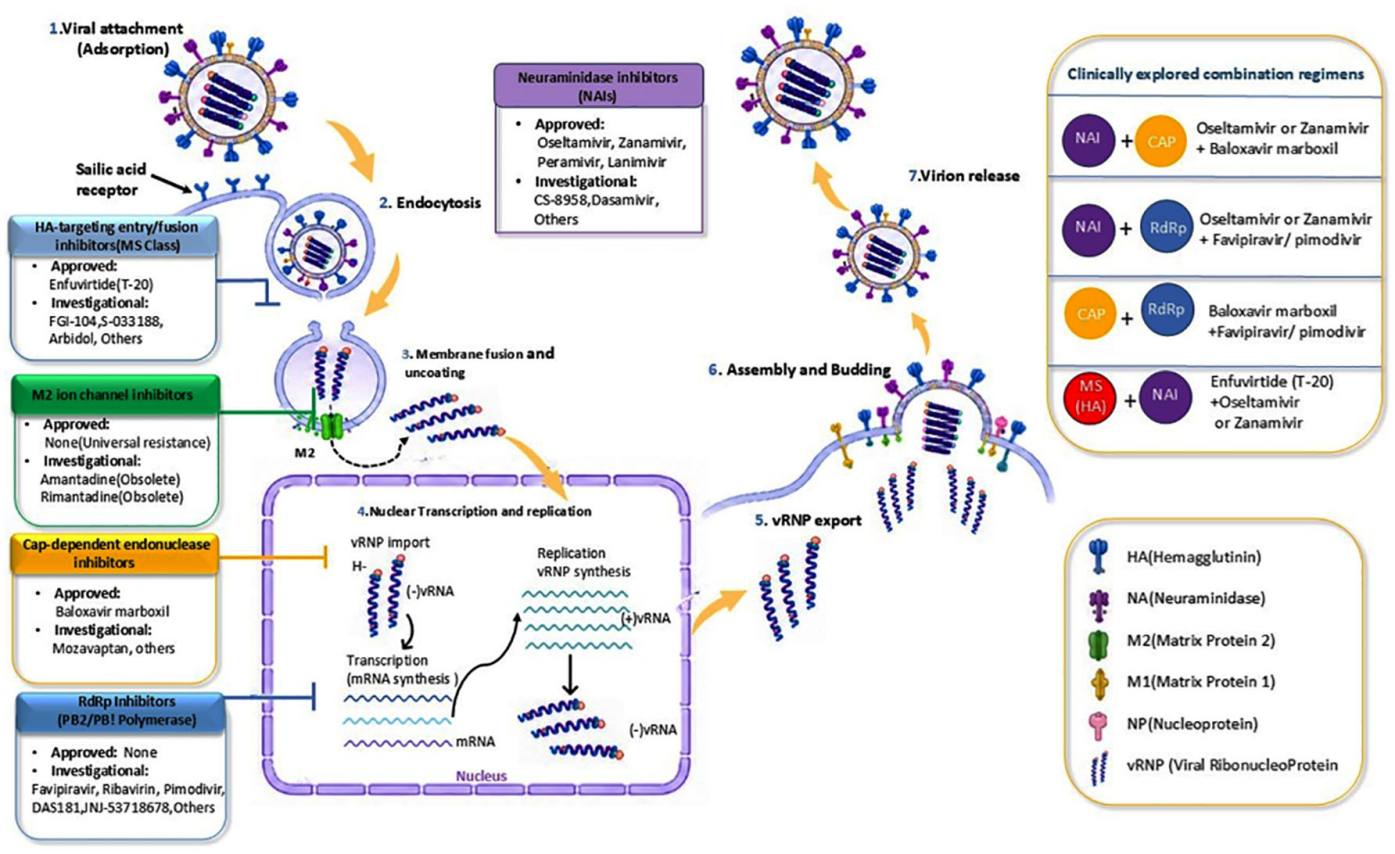
Influenza virus replication cycle and sites of antiviral drug action. The major stages of viral replication are shown, including cell adhesion, endocytosis, viral uncoating, nuclear transcription and replication, vRNP export, and virion budding and release. Drug classes are annotated at their respective sites of action. Bold entries indicate approved agents; non-bold entries indicate investigational compounds. The clinically explored combination regimen are summarized in the top-right panel. NAI, neuraminidase inhibitor; RdRp, RNA-dependent RNA polymerase; HA, hemagglutinin; M2, matrix protein 2; vRNP, viral ribonucleoprotein; MS, membrane-stabilizing (HA fusion inhibitor class).

The development of influenza antivirals has occurred in distinct therapeutic waves: M2 ion channel inhibitors (now clinically obsolete due to universal S31N resistance), neuraminidase inhibitors (still first-line but with a narrow therapeutic window and resistance vulnerabilities), and most recently, cap-dependent endonuclease inhibitors led by baloxavir marboxil, which offer a single-dose regimen targeting the viral polymerase but with treatment-emergent resistance at the PA-I38 residue (Hayden et al., 2018). Each successive class has addressed limitations of its predecessor while introducing new ones. This review aims to critically evaluate the full breadth of the influenza antiviral arsenal across five domains: (1) the established neuraminidase inhibitor class alongside emerging cap-dependent endonuclease inhibitors and other polymerase-targeting agents; (2) the molecular basis and clinical implications of antiviral resistance across all approved drug classes; (3) the translational status of host-directed therapies, biologics, and repurposed drugs as adjuncts or alternatives to direct-acting antivirals; (4) the specific therapeutic preparedness gaps exposed by the ongoing H5N1 threat; and (5) the next-generation antiviral platforms and policy priorities needed to close these gaps. In doing so, it identifies where the current evidence base is sufficient to guide clinical practice, and where it remains too limited to do so (**Figure 1**).

## 2 Current clinical standards: neuraminidase inhibitors

### 2.1 Neuraminidase as a therapeutic target in the influenza life cycle

NA has been validated as a therapeutic target since its activity is crucial in the late phases of the influenza life cycle. Inhibiting neuraminidase prevents cleavage of terminal sialic acid residues, which keeps virions at the cell surface and slows the spread of infection. Molecular and structural studies have revealed that the conserved catalytic site of NA across influenza A and B allows for the rational design of small-molecule inhibitors which competitively block its active site (von Itzstein, 2007).

**Figure 2:**
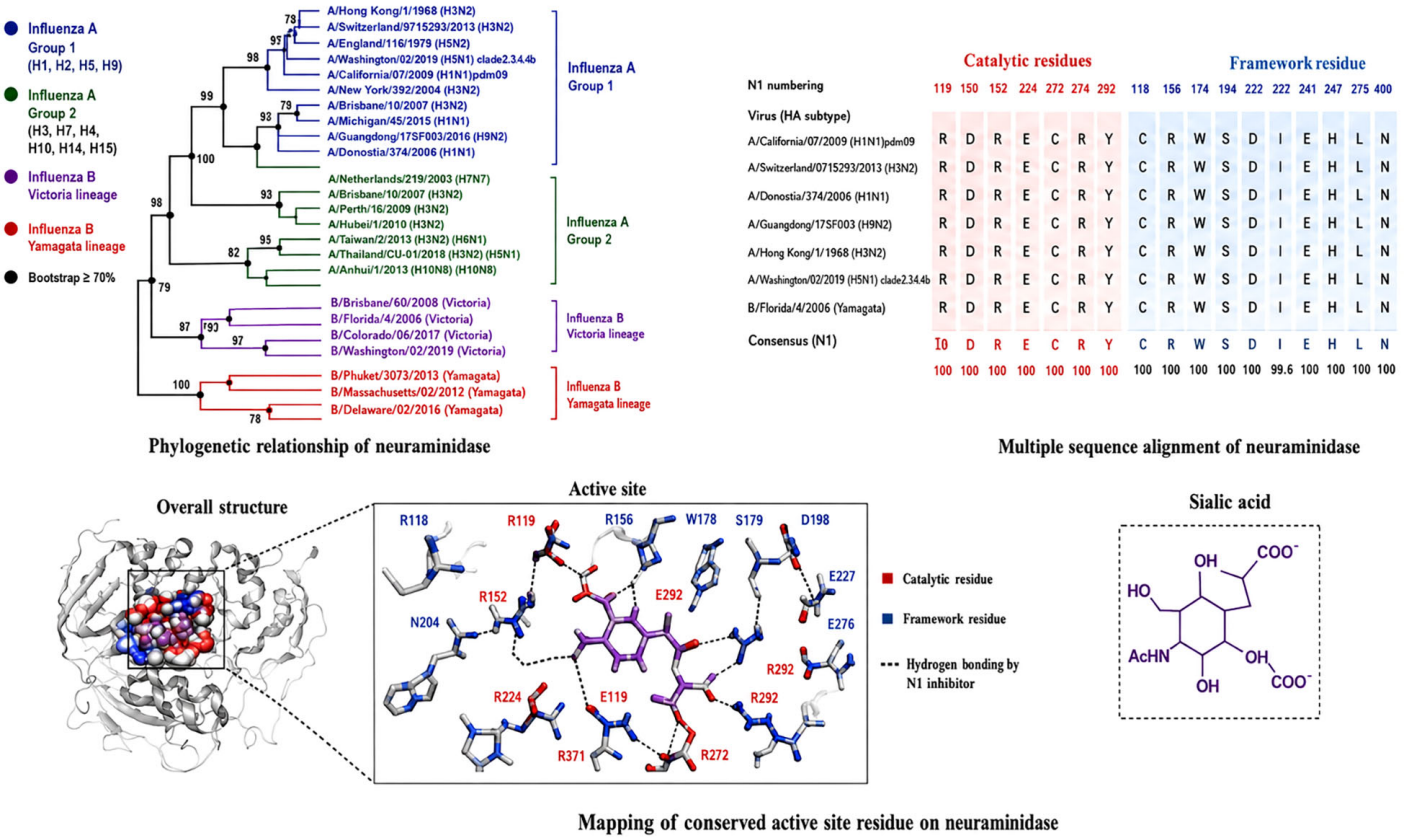
Phylogenetic relationships, active site conservation, and structural mapping of influenza neuraminidase. Upper left: Maximum-likelihood phylogenetic tree of influenza neuraminidase sequences across Group 1 (H1, H2, H5, H9) and Group 2 (H3, H7, H4, H10, H14, H15) influenza A subtypes and influenza B Victoria and Yamagata lineages. Bootstrap values ≥70% are shown at key nodes. Upper right: Multiple sequence alignment of neuraminidase active site residues across representative influenza A and B strains. Catalytic residues (R118, D151, R152, R224, E276, R292, R371, Y406; highlighted in red) and framework residues (E119, R156, W178, S179, D198, I222, E227, H274, E277, N294, E425; highlighted in blue) show 100% or near-complete conservation across all subtypes. Lower left: Three-dimensional structure of the neuraminidase active site with all catalytic residues labeled (red: R118, D151, R152, R224, E276, R292, R371, Y406) and framework residues (blue: E119, R156, W178, S179, I222, E277, N294), showing the binding pose of a neuraminidase inhibitor (purple sticks) in contact with the conserved active site. Dashed lines indicate hydrogen bonding and electrostatic interactions with active site residues. Dashed lines indicate key molecular contacts. Lower right: Chemical structure of sialic acid (N-acetylneuraminic acid), the natural NA substrate mimicked by all licensed neuraminidase inhibitors.

The neuraminidase active site consists of 19 conserved residues in two functional groups. The eight catalytic residues (R118, D151, R152, R224, E276, R292, R371, and Y406) are directly in contact with the sialic acid substrate and are absolutely conserved among all nine NA subtypes because of their critical function in the enzyme. There are 11 framework residues (E119, R156, W178, S179, D/N198, I222, E227, H274, E277, N294, and E425) that form the structural scaffold positioning the catalytic residues and are similarly conserved across all nine NA subtype. All four licensed NAIs make contacts with residues from both groups, which accounts for their broad activity across influenza A subtypes and influenza B. H275Y resistance mutation changes the bulkier tyrosine close to framework residue H274 and catalytic residue E276, causing steric displacement of the carboxylate group of oseltamivir, but not the smaller zanamivir scaffold (**Figure 2****).**

### 2.2 Mechanism of neuraminidase inhibition

Each clinically used NAI has a similar competitive mechanism but differs in chemical structure and pharmacokinetic properties. For example, oseltamivir is given orally as a prodrug that is converted to oseltamivir carboxylate, which binds to the neuraminidase active site with great affinity (Moscona, 2005). Zanamivir is administered by inhalation and acts as a powerful transition-state analog (Moscona, 2005). Peramivir and laninamivir share the NA active site, but differ in their routes of administration and duration of action (Leang et al., 2014; Alame et al., 2016; Roosenhoff et al., 2020).

**Figure 3:**
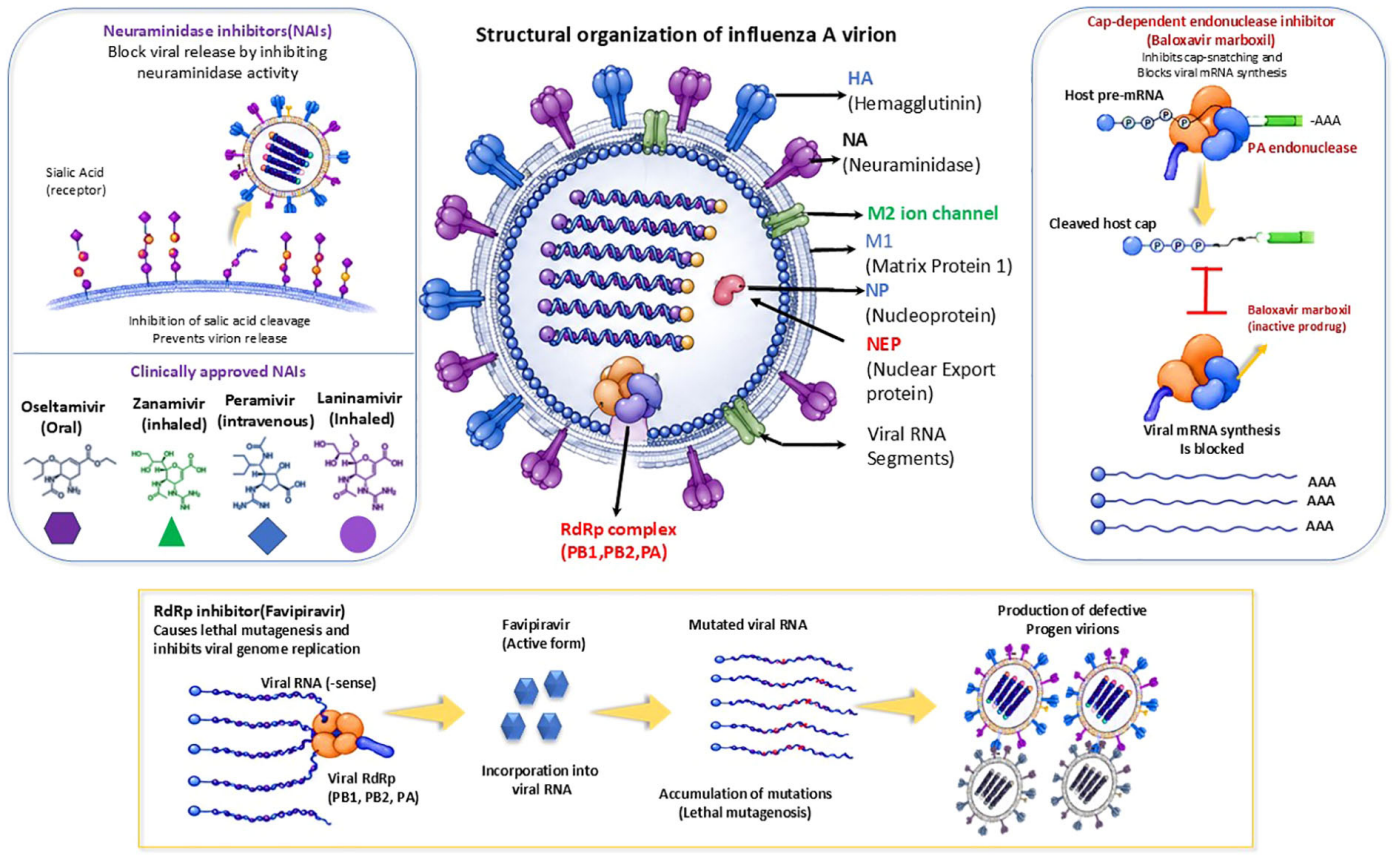
Structural organization of the influenza A virion and mechanisms of action of approved antiviral drug classes. The major structural components of the virion are labeled, including the surface glycoproteins hemagglutinin (HA) and neuraminidase (NA), the M2 ion channel, matrix protein M1, nucleocapsid protein NP, nuclear export protein NEP, and the segmented negative-sense RNA genome associated with the trimeric RNA-dependent RNA polymerase complex (PB1, PB2, PA). Inset panels illustrate the mechanism of neuraminidase inhibitors at the viral entry and release stage, favipiravir-mediated disruption of genome replication via lethal mutagenesis, and baloxavir marboxil inhibition of cap-snatching via PA endonuclease blockade.

As neuraminidase drives viral egress rather than intracellular replication, NAIs are most effective in the first 48 hours of infection, when active virion release is at its peak (Gillissen and Höffken, 2002). These drugs reduce viral load, shorten the duration of the disease, and minimize transmissibility by blocking neuraminidase; however, the degree of clinical effect can vary depending on host and viral factors (CDC, 2026a) (**Figure 3**).

### 2.3 First-generation NAIs

#### 2.3.1 Oseltamivir (Tamiflu)

Oseltamivir phosphate, commercially known as Tamiflu, is an ethyl ester prodrug that is rapidly hydrolyzed by carboxylesterases in the intestinal wall and liver to its active metabolite; oseltamivir carboxylate, which binds to the conserved catalytic site of influenza neuraminidase (Moscona, 2005). The drug was developed using structure-based design to mimic the transition state of the natural substrate, using a hydrophobic side chain that improves oral bioavailability compared with earlier neuraminidase inhibitors (von Itzstein et al., 1993). According to a meta-analysis of 11 randomized controlled trials, early administration reduces the length of illness by around 1–2 days and may reduce the risk of lower respiratory tract complications (Hernán and Lipsitch, 2011). The most frequently reported adverse effects are gastrointestinal, specifically nausea and vomiting, which are generally mild and temporary (Moscona, 2005).

#### 2.3.2 Zanamivir (Relenza)

**Figure 4:**
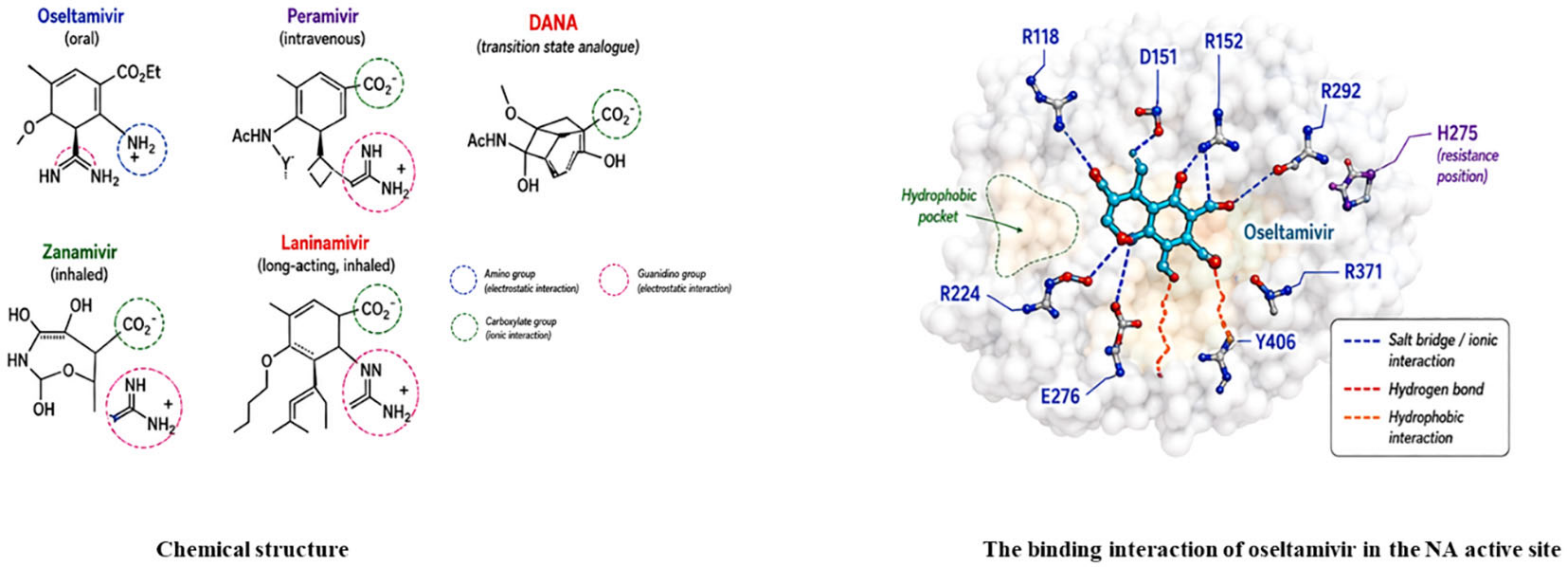
Chemical structures and binding interactions of approved neuraminidase inhibitors. Left panel: Chemical structures of oseltamivir (oral), zanamivir (inhaled), peramivir (intravenous), laninamivir (long-acting inhaled), and the transition-state analog DANA from which zanamivir was rationally derived. Key pharmacophoric groups are annotated: carboxylate groups (green dashed circles) providing ionic interactions with the conserved arginine triad; amino groups (blue dashed circles) and guanidino groups (pink dashed circles) providing electrostatic interactions with framework and catalytic residues. Right panel: Three-dimensional binding pose of oseltamivir within the neuraminidase active site. Salt bridges and ionic interactions (blue dashed lines), hydrogen bonds (red dashed lines), and hydrophobic interactions (orange dashed lines) with conserved active site residues are shown. H275 (purple) marks the resistance position where H275Y substitution causes steric displacement of the oseltamivir carboxylate without affecting zanamivir’s guanidino group. The hydrophobic pocket (green shading) accommodates oseltamivir’s pentyl ether side chain, explaining its superior oral bioavailability compared to the polar zanamivir scaffold. the binding interaction panel illustrates oseltamivir binding at the neuraminidase active site; equivalent interactions occur for other NAIs at the same conserved residues, with pharmacophore group-specific contacts as described in the text.

Zanamivir was the first neuraminidase inhibitor to be clinically approved. It was designed as a structural analog of 2,3-dehydro-2-deoxy-N-acetylneuraminic acid (DANA) by rational structure-based drug design (von Itzstein et al., 1993). The highly polar nature of zanamivir severely restricts oral bioavailability, necessitating inhalation as a dry powder for outpatient use or intravenous administration in hospitalized patients unable to use the inhaled formulation (Cass et al., 1999). Zanamivir is generally well tolerated, but since it is inhaled, patients with underlying reactive airway disease should exercise caution due to the possibility of bronchospasm (Cheer and Wagstaff, 2002) (**Figure 4**).

### 2.4 Second-generation and long-acting NAIs

To address these limitations, agents have been developed which are referred to as second generation: peramivir has a scaffold of cyclopentane, which has a slow off-rate from the NA active site, resulting in intravenous administration in a single dose; laninamivir is a prodrug based on a scaffold with extended airway tissue retention, which allows for a single inhaled dose to provide inhibitory concentrations. Second-generation agents were developed to address two unmet needs: providing a parenteral treatment option for patients unable to use oral or inhaled formulations, and eliminating the five-day adherence burden in a disease that typically peaks before day three of illness.

#### 2.4.1 Peramivir (Rapivab)

Peramivir (BCX-1812; Rapivab) is a cyclopentane-scaffold neuraminidase inhibitor, structurally distinct from the cyclohexene scaffold of oseltamivir and the dihydropyran scaffold of zanamivir. The pharmacokinetics for single-dose intravenous administration are the unusual slow off-rate from the neuraminidase active site, resulting from this scaffold’s geometry (Kohno et al., 2010; Marty et al., 2017). Because peramivir shares the same carboxylate-mediated contact at E276 as oseltamivir, it carries the same cross-resistance to H275Y-bearing A(H1N1) strains (Kohno et al., 2010; Marty et al., 2017).

Peramivir is excreted unchanged in the urine via active tubular secretion and does not require hepatic bioactivation, which means that it can be used in patients with hepatic impairment or gastrointestinal dysfunction in which the absorption of its oral prodrug may be impaired (Marty et al., 2017). In hospitalized patients with severe influenza, a separate Phase 3 study established that once-daily peramivir 600 mg for five days provided virological and clinical outcomes comparable to intravenous zanamivir and twice-daily oseltamivir, supporting its use in this setting (Marty et al., 2017). Due to the renal elimination pathway, renal monitoring is recommended in patients with pre-existing renal impairment (Marty et al., 2017).

#### 2.4.2 Laninamivir (Inavir)

Laninamivir octanoate (CS-8958; Inavir) is a long-chain octanoyl ester prodrug of laninamivir: the parent compound is structurally similar to zanamivir with a significantly increased lipophilicity because of the long-chain octanoyl ester at the C3 position of the dihydropyran ring. Unlike zanamivir and oseltamivir, which are rapidly removed from the respiratory epithelium and must be administered multiple times, the octanoate ester forms a depot and is hydrolyzed to release the active drug, laninamivir, locally from airway tissue (Kiso et al., 2010; Sugaya and Ohashi, 2010). Like zanamivir, laninamivir’s guanidino-based pharmacophore retains full activity against H275Y-resistant strains, which displace the oseltamivir carboxylate but not the guanidino contact residues (von Itzstein, 2007; Sugaya and Ohashi, 2010).

Human pharmacokinetic studies have shown that the concentration of laninamivir in nasal epithelial cells and in bronchial tissue is maintained above the IC50 for representative influenza A and B strains for up to 240 hours, as demonstrated in human bronchial epithelial cell culture models, after a single dose of 80 mg administered by inhalation (no repeated dosing is required for the replication cycle of acute influenza infection) (Sugaya and Ohashi, 2010).

A clinically important subgroup finding was a significant virological and clinical benefit of laninamivir over oseltamivir in children infected with oseltamivir-resistant A(H1N1) strains carrying H275Y, where oseltamivir lost efficacy but laninamivir retained full activity, directly confirming in clinical practice the pharmacophore-based cross-resistance profile differences predicted by structural analyses (Sugaya and Ohashi, 2010).

Given the inhaled dry powder drug delivery system, the major safety concern with laninamivir octanoate is bronchospasm in patients with reactive airway disease, such as asthma or COPD, which is a class effect of all inhaled NAIs, including zanamivir (Kashiwagi et al., 2012). The safety study of laninamivir octanoate conducted in Japan included more than 4,800 patients, and no serious adverse events that were considered to be related to the drug were identified. As laninamivir is approved only in Japan and China, the global post-marketing safety database remains limited (Kashiwagi et al., 2012). (**Table 1**).

**Table 1:** Comprehensive comparison of approved and investigational influenza antivirals.

Neuraminidase inhibitors (NAIs)							
Drug (brand)	Drug class	Primary target	Mechanism of action	Spectrum	Route	Dose/regimen	Pivotal clinical evidence
Oseltamivir (Tamiflu)	1st-Gen NAI	Neuraminidase (NA) N1/N2 active site	Competitive inhibition of terminal sialic acid cleavage; prevents virion release from host cell surface	Flu A + B	Oral (prodrug)	75 mg BID × 5 d (Tx) 75 mg QD × 10 d (PEP)	(Nicholson et al., 2000; Muthuri et al., 2014)
Zanamivir (Relenza)	1st-Gen NAI	Neuraminidase (NA) N1/N2 active site	Transition-state analog of sialic acid; competitive active-site inhibition	Flu A + B	Inhaled (DPI)	10 mg BID × 5 d (Tx) 10 mg QD × 10 d (PEP)	(The MIST (Management of Influenza in the Southern Hemisphere Trialists) Study Group, 1998; Cooper et al., 2003)
Peramivir (Rapivab)	2nd-Gen NAI	Neuraminidase (NA) active site	Cyclopentane scaffold; competitive NA inhibition; slow off-rate allows single dose IV administration	Flu A + B	IV infusion	600 mg single dose (Tx) 600 mg QD ≥5 d (hospitalized)	(Kohno et al., 2010; Marty et al., 2017)
Laninamivir (Inavir)	Long-Acting NAI	Neuraminidase (NA) active site	Prodrug hydrolyzed in airways; prolonged retention in respiratory epithelium; single-dose treatment	Flu A + B	Inhaled (DPI)	80 mg single inhaled dose (adults)	(Sugaya and Ohashi, 2010; Kashiwagi et al., 2012)
Polymerase-targeting antivirals							
Baloxavir Marboxil (Xofluza)	Cap-Endonuclease Inhibitor (CENI)	PA subunit Endonuclease domain	Metal chelation of PA active site (His41/Glu80/Asp108/Glu119); blocks cap-snatching; inhibits viral mRNA synthesis initiation	Flu A + B; H5N1; H7N9; NAI-resistant	Oral (prodrug)	40 mg single dose (<80 kg) 80 mg single dose (≥80 kg)	(Hayden et al., 2018; Kumar et al., 2022)
Favipiravir (Avigan^®^)	Nucleoside Analog (PB1 Inhibitor)	PB1 RdRp catalytic subunit	Converted to favipiravir-RTP by cellular enzymes; incorporated into vRNA causing lethal mutagenesis (C→U, G→A transitions) via error catastrophe	Flu A + B; H5N1; broad RNA virus activity	Oral	1600 mg BID Day 1 then 600 mg BID Days 2–5	(Hayden et al., 2022)
Pimodivir (VX-787)	Non-nucleoside PB2 Inhibitor	PB2 cap-binding domain (CBD)	Binds m7G cap-binding pocket of PB2; blocks cap delivery to PA for cleavage; inhibits viral mRNA initiation upstream of PA endonuclease	Flu A only (not Flu B)	Oral	600–900 mg BID (Phase 2 dosing)	(Finberg et al., 2019)
Direct-acting antivirals - non-polymerase targets							
Umifenovir (Arbidol^®^)	HA Fusion Inhibitor	HA2 stalk trimer interface	Binds HA stem hydrophobic pocket; stabilizes pre-fusion trimer; blocks pH-triggered HA refolding required for endosomal membrane fusion	Flu A + B; broad antiviral (CYP3A4 substrate)	Oral	200 mg TID × 5 days (Russia/China approved)	(Kiselev et al., 2015; Feng et al., 2022)
Amantadine/Rimantadine	M2 Ion Channel Blocker (Adamantane)	M2 proton channel (Flu A)	Occludes M2 pore; blocks proton conductance required for endosomal acidification, M1-RNP dissociation, and viral uncoating	Flu A only (NOT Flu B)	Oral	100 mg BID (amantadine) 100 mg BID (rimantadine)	(Davies et al., 1964; Hayden et al., 1991)
Host-directed therapies (HDTs) - selected agents							
Nitazoxanide (Alinia^®^)	Thiazolide Host-Directed	HA glycosylation pathway (ER→Golgi)	Blocks HA transit from ER to Golgi; prevents HA trimerization and surface incorporation; activates PKR/eIF2α integrated stress response	Flu A + B; broad antiviral; resistance-resistant	Oral	600 mg BID × 5 days	(Haffizulla et al., 2014)
Zapnometinib (ATR-002)	MEK1/2 Inhibitor Host-Directed	RAF/MEK/ERK cascade	Blocks ERK-mediated nuclear export of vRNP; reduces viral replication without directly targeting viral proteins; synergistic with NAIs	Flu A + B; SARS-CoV-2 (preclinical)	Oral	Phase II dosing (TBD)	(Schreiber et al., 2022)
DAS181 (Fludase)	Sialidase Fusion Protein (HDT)	Airway epithelial sialic acid receptors	Recombinant human lung sialidase; removes α2,3- and α2,6-linked SA from airway epithelia; eliminates HA attachment sites; mechanism entirely independent of NA activity	Flu A + B; Parainfluenza; NAI-resistant strains	Inhaled (nebulized)	Phase 2 dosing (Moss et al., 2012)	(Chemaly et al., 2021)

### 2.5 Clinical efficacy and limitations

The clinical efficacy of NAIs has been extensively studied but remains contested. The most consistently documented advantage of NAIs is a slight reduction in the duration of influenza symptoms in otherwise healthy individuals. A Cochrane systematic review and meta-analysis of published and regulatory trial data found that oseltamivir reduced time to symptom relief in previously healthy adults by 16.8 hours compared with placebo, a result that is statistically significant but of modest absolute clinical magnitude in immunocompetent individuals (Jefferson et al., 2014).

Despite this debate, the effect of early treatment with NAIs has shown more compelling results in certain high-risk patient groups. A large retrospective cohort study and several observational studies have established that early oseltamivir treatment significantly decreases the occurrence of pneumonia, hospitalization, and mortality in patients with severe influenza infections, including those caused by pandemic strains of H1N1 (Whitley et al., 2001). A pooled patient data analysis from 78 studies on over 29,000 patients with influenza A(H1N1)pdm09 showed that early treatment with NAIs significantly reduced the risk of mortality from this infection (Muthuri et al., 2014). The reduction in mortality risk was found to be greatest if the treatment was given within 48 hours of onset of symptoms (Muthuri et al., 2014). Even if treatment is given after 48 hours, there is still a benefit in critically ill and hospitalized patients (Muthuri et al., 2014).

In children, oseltamivir additionally reduces the incidence of otitis media as an influenza complication, and both agents achieve 70-90% prophylactic efficacy in household and institutional settings (Hayden et al., 1999; Whitley et al., 2001; Heinonen et al., 2010).

Despite their well-established use in the management of influenza, NAIs have a number of significant drawbacks that restrict their therapeutic potential.

#### 2.5.1 Time dependency of efficacy

NAIs have been shown to have their major antiviral effect during the period of active viral replication and egress. As neuraminidase inhibition does not directly inhibit intracellular replication, efficacy is reduced if treatment is delayed beyond 48 hours of the onset of symptoms in uncomplicated influenza (Jefferson et al., 2014). This represents a major logistical difficulty in treatment, as this period may have elapsed before the patient presents for assessment and diagnosis.

#### 2.5.2 Modest effect size in healthy adults

The absolute benefit of NAIs for otherwise healthy outpatients with uncomplicated influenza appears to be modest. In the Cochrane review, Jefferson et al. found that there was little convincing evidence that oseltamivir reduces the risk of complications such as pneumonia and hospitalization for the general adult population, and expressed concern about the reliability of some of the trial data (Jefferson et al., 2014). This led to a re-evaluation of the use of stockpiled oseltamivir for pandemic influenza preparedness.

#### 2.5.3 Antiviral resistance

NAI resistance has been increasingly recognized as a threat to the continued effectiveness of this class of drugs. Resistance to NAI has been mediated by mutations in the NA gene, including the H275Y mutation in N1 neuraminidase, which confers high-level resistance to oseltamivir but maintains susceptibility to zanamivir (Hurt et al., 2009; Moscona, 2009). Global circulation of oseltamivir-resistant H1N1 was noted between 2007 and 2009. This experience highlighted the potential for widespread circulation of NAI-resistant viruses in the absence of significant drug pressure. This has sparked concern regarding the continued effectiveness of oseltamivir as a monotherapy agent (Moscona, 2009). Current circulating strains of influenza have maintained susceptibility to oseltamivir; nonetheless, continued monitoring of resistance emergence is warranted, especially in the presence of drug pressure from widespread prophylactic and therapeutic use (Hurt et al., 2009).

#### 2.5.4 Limitations in severe and hospitalized disease

Although observational data support the use of NAIs in the hospital and among severely ill patients, there is minimal evidence from randomized controlled trials in these situations. Studies including the IRIS program demonstrated that by the time of hospitalization, active viral replication may already be waning. It limits the additional virological and clinical benefit of NAI therapy at this stage. Furthermore, randomized trials evaluating higher-dose oseltamivir regimens in hospitalized patients failed to demonstrate superiority over standard dosing (Hayden et al., 2022; Schreiber et al., 2022).

Interpretation of observational data is complicated by confounding by indication, where patients receiving early treatment may differ systematically from those not receiving early treatment.

#### 2.5.5 Pharmacokinetic and tolerability constraints

Oseltamivir is the most commonly used NAI but it requires hepatic metabolism to its active metabolite and has gastrointestinal side effects, including nausea and vomiting, among a significant proportion of patients (Moscona, 2005). Zanamivir, being an inhaled formulation, has limitations among patients with underlying obstructive airway disease and among patients unable to use the device, such as young children and intubated patients (The MIST (Management of Influenza in the Southern Hemisphere Trialists) Study Group, 1998). Although peramivir and laninamivir provide more options for different routes of administration and different schedules, the data supporting the use of these newer agents over the older agents for patient outcomes are lacking for most patient populations (Kiso et al., 2010; Kohno et al., 2010).

#### 2.5.6 Lack of activity against non-influenza respiratory viruses

NAIs are very specific to the neuraminidase enzyme of the Influenza virus and have no activity against any other respiratory viruses, including SARS-CoV-2, RSV, and parainfluenza. This level of specificity makes the early and accurate diagnosis of influenza infection a requirement before the initiation of antiviral treatment, which may not always be possible, especially in resource-constrained settings (Hayden, 2006).

Although NAIs remain the standard of care for the treatment and prophylaxis of influenza, the limitations of NAIs highlight the urgent need for the development of the next generation of antivirals that are active against influenza, have a wider therapeutic window, are less likely to develop resistance, and offer greater clinical benefit for severe influenza disease.

Across the NAI class, successful agents share three features: a structurally conserved target under strong functional constraint, a mechanism acting at viral egress rather than replication, and a pharmacokinetic profile suitable for outpatient use. Those agents that have failed clinically share the pattern of having a zero-fitness-cost resistance mutation (S31N) that has spread globally without drug pressure, whereas the threat of resistance from NAIs is more specific, with two mutations (H275Y and R292K) that require permissive genetic backgrounds to reach epidemic fitness. The one big structural flaw in the whole class of NAIs is the time-dependent effectiveness of the therapy; clinical benefit is seen in the first 48 hours of NAI therapy, but most patients present after this period. This limitation is directly solved by any next generation agent targeting a different stage of the replication process, or a combination approach that would prolong the therapeutic window.

## 3 Polymerase-targeting antivirals: expanding arsenal

### 3.1 The influenza polymerase complex as a therapeutic hub

The RNA-dependent RNA polymerase complex of Influenza virus is a trimeric enzyme that consists of three different subunits, named the polymerase basic protein 1 (PB1), the polymerase basic protein 2 (PB2), and the polymerase acidic protein (PA). Together, these three subunits transcribe and replicate the viral genome in the host cell nucleus, making the polymerase complex a high-priority antiviral target (Pflug et al., 2017).

The PB1 subunit of the viral polymerase complex is the catalytic core of the enzyme complex, which has RNA-dependent RNA polymerase activity. It has the classic GDD motif found in RNA polymerases and is responsible for nucleotide incorporation during both genome replication and mRNA synthesis (Biswas and Nayak, 1994). It functions as the heterotrimer’s core scaffold and interacts with PA through its N-terminus and PB2 through its C-terminus (Sugiyama et al., 2009).

PB2, a 759-amino-acid subunit, is principally important for detecting and binding the 5’ 7-methylguanosine (m7G) cap structures on host pre-mRNAs. This cap-binding activity, which is confined to a specific cap-binding domain between residues 318-483, is required for the cap-snatching mechanism that initiates viral mRNA production (Guilligay et al., 2008; Resa-Infante et al., 2011). PB2 also has a nuclear localization signal and is crucial for determining host range and virulence, especially through residue 627, where lysine (627K) is associated with improved human replication and mammalian adaptability (Subbarao et al., 1993).

The N-terminal domain of PA (PA-N, residues 1-209) has a Mn2+-dependent endonuclease activity necessary for cap-snatching, and its C-terminal domain enables contact with N-terminus of PB1 (Dias et al., 2009; Yuan et al., 2009). In order to produce capped RNA primers for viral mRNA transcription, the PA endonuclease domain cleaves host pre-mRNAs 10–13 nucleotides downstream of the 5′ cap using a two-metal-ion catalytic mechanism (DuBois et al., 2012). PA has a C-terminal unique fold that facilitates PB1 binding via a highly conserved interface, making this protein-protein interaction a potential target for disruption (He et al., 2008).

The cap-snatching process is a distinct viral approach that differentiates influenza transcription from host cell mRNA production. In this mechanism, the PB2 subunit attaches to the 5′ cap of newly formed host pre-mRNAs, and the PA endonuclease cleaves the capped oligonucleotide fragment 10–13 nucleotides downstream. The capped primer is subsequently employed by PB1 to initiate viral mRNA synthesis from the viral RNA (vRNA) template (Reich et al., 2014; Te Velthuis and Fodor, 2016) This process is absent from host cell biology, making it an antiviral target with an inherently favorable therapeutic index. The whole trimeric complex assembles in the cytoplasm prior to nuclear import, where it associates with viral ribonucleoprotein (vRNP) complexes that also contain the nucleoprotein (NP) and the segmented viral genome (Eisfeld et al., 2015).

**Figure 5:**
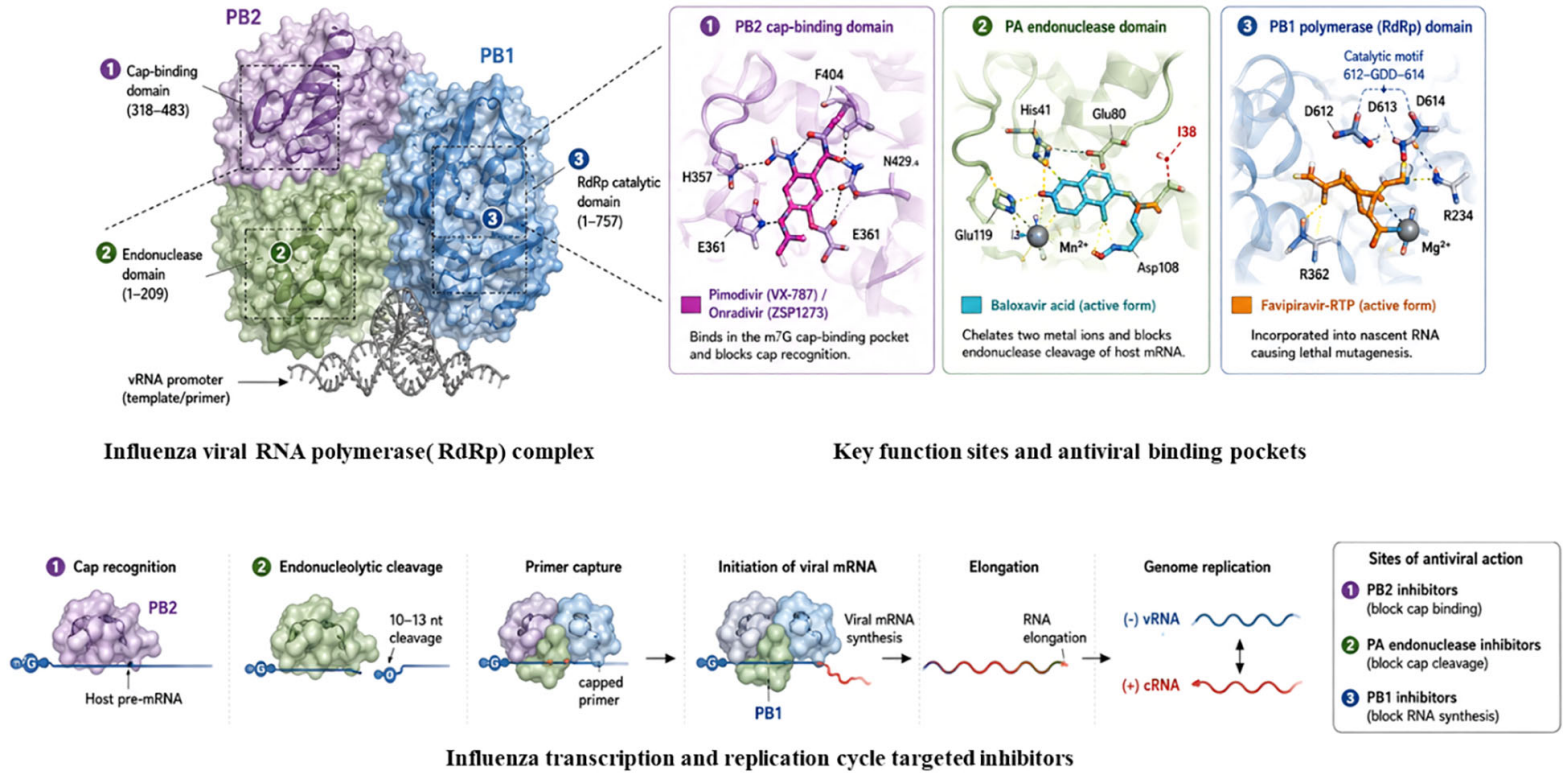
Structure of the influenza RNA-dependent RNA polymerase (RdRp) complex and antiviral binding pockets. Top: the trimeric PA-PB1-PB2 complex with three drug target domains annotated (the PB2 cap-binding domain, the PA endonuclease domain, and the PB1 catalytic domain), alongside close-up views of each pocket showing pimodivir/onradivir at the PB2 cap-binding site, baloxavir acid chelating the PA two-metal-ion active site (with the I38 resistance residue marked), and favipiravir-RTP incorporated at the PB1 catalytic GDD motif. Bottom: the cap-snatching mechanism, from PB2-mediated host cap recognition through PA endonucleolytic cleavage to PB1-driven viral mRNA synthesis, with the site of action of each inhibitor class indicated.

Recent studies by cryo-electron microscopy and X-ray crystallography have led to a better structural understanding of the trimeric polymerase complex and its various functional states, including transcription initiation, elongation, and replication, greatly advancing drug design against these targets (Pflug et al., 2014; Thierry et al., 2016) (**Figure 5**).

### 3.2 PA-targeting inhibitors

#### 3.2.1 Cap-dependent endonuclease inhibitors

The cap-dependent endonuclease (CEN) domain of PA, represents a clinically validated target for antiviral development. CENI act by interfering with the metal-dependent catalytic activity required for host mRNA cleavage.

The initial efforts in CENI discovery focused on natural product derivatives and flutimide analogs. Flutimide (a fungal secondary metabolite structurally belonging to the diketoacid class) was one of the first compounds reported as a PA endonuclease inhibitor (Tomassini et al., 1994). Flutimide established chelation as a mechanism for CEN inhibition. However, subsequent SAR evaluations of flutimide derivatives led to the identification of diketoacids and hydroxypyridinones as potent chelators of metal ions essential for CEN activity. Diketoacids and hydroxypyridinones are effective CENIs in cell culture and animal models (Baughman et al., 2012). Structural understanding of these initial compounds formed the basis for developing even more potent and metabolically stable CENI, leading to the clinical licensure of baloxavir marboxil (Noshi et al., 2018).

#### 3.2.2 Baloxavir marboxil

Baloxavir marboxil (S-033188; Xofluza^®^) is an orally bioavailable prodrug that is rapidly metabolized in the liver to its active form, baloxavir acid (S-033447), upon intestinal absorption (Omoto et al., 2018; Fukao et al., 2019). Baloxavir acid is a first-in-class selective inhibitor of the PA endonuclease subunit of the influenza virus polymerase, targeting the same catalytic domain described in described in the polymerase complex overview above. Baloxavir is differentiated from neuraminidase inhibitors by its distinct mechanism of action, targeting the viral polymerase rather than neuraminidase. Baloxavir marboxil was developed by Shionogi & Co., with commercialization rights outside Japan subsequently licensed to Roche/Genentech. It was approved in Japan and the US in 2018 (Hayden et al., 2018).

High-resolution structures of baloxavir acid in complex with PA have confirmed direct interaction with the catalytically active residues His41, Glu80, Asp108, and Glu119. Consequently, viral mRNA synthesis is blocked in its initiation step, thereby eliminating viral replication without direct interference in the polymerase replication function (Noshi et al., 2018).

From the clinical perspective, baloxavir marboxil has shown comparable efficacy to oseltamivir in the reduction of time to symptom relief and the faster reduction of viral load, as evidenced in the Phase 3 CAPSTONE-1 and CAPSTONE-2 trials (Hayden et al., 2018; Ison et al., 2020). A single oral dose of baloxavir marboxil (40 mg for patients with a weight of ≤80 kg, and 80 mg for patients with a weight of >80 kg) is sufficient for the systemic exposure required for the suppression of influenza virus replication for the duration of the illness, thereby showing a substantial advantage over the five-day regimen of oseltamivir (Hayden et al., 2018; Watanabe et al., 2019). Baloxavir marboxil has been approved for the treatment of adults as well as for children aged ≥1 year and the compound has also been proven to have the potential for use in high-risk groups, including the elderly and patients with co-morbidities (Baker et al., 2020; Ison et al., 2020). Plus, baloxavir marboxil is active against influenza A viruses, including the H5N1 and H7N9 strains of the avian flu virus, as well as the influenza B virus (Mishin et al., 2019). Baloxavir marboxil is also active against oseltamivir-resistant strains of the influenza virus that have the H275Y mutation in the neuraminidase protein (Mishin et al., 2019).

#### 3.2.3 PA-I38X resistance

Despite the therapeutic efficacy of baloxavir, the development of resistant variants such as those harboring I38 substitutions in the PA subunit has sparked concerns regarding the long-term efficacy of the drug. The I38T substitution is the most common resistance-associated variant (RAV), although other substitutions such as I38F, I38M, and I38L have also been identified (Checkmahomed et al., 2020; Uehara et al., 2020). In the Phase 3 CAPSTONE-1 randomized controlled trial (Hayden et al., 2018), PA-I38T or PA-I38M substitutions emerged in 2.2% of A(H1N1)pdm09-infected recipients and 9.7% of A(H3N2)-infected baloxavir recipients. Separately, in a Japanese open-label pediatric study of children aged 1–11 year (Hirotsu et al., 2020), PA/I38T/M variants emerged in 23.4% of treated patients. The molecular mechanisms underlying PA-I38X resistance, fitness implications, and surveillance data are discussed in detail in Section 8.

### 3.3 Next-generation cap-dependent endonuclease inhibitors

Baloxavir marboxil is the only CENI that has wide regulatory approval internationally, but a number of structurally related PA endonuclease inhibitors have recently been developed to late-stage clinical development and received regulatory approval in China (Wang et al., 2025; Wang et al., 2025; Yang et al., 2025). It provides an important proof-of-concept to the next generation of single-dose polymerase inhibitors with potentially better resistance profiles.

Suraxavir marboxil (GP681) is an oral prodrug that is transformed to suraxavir acid, which is a selective PA endonuclease inhibitor with a different chemical scaffold than baloxavir acid (Wang et al., 2025). In January 2025, a Phase 3 randomized controlled trial in the journal Nature Medicine showed that a single dose of suraxavir marboxil reduced the time to symptom alleviation significantly (42.0 vs. 63.0 hours; p = 0.002) in patients with uncomplicated influenza (Wang et al., 2025). Importantly, treatment-emerging PA-I38T resistance variants were identified in only 0.7-0.9% of treated patients, which is far lower than the 2.2-9.7% of baloxavir in the CAPSTONE-1 trial (Hayden et al., 2018; Wang et al., 2025), indicating that there might be a slightly higher genetic barrier to resistance emergence in the drug-PA interaction. Suraxavir has already been granted regulatory approval in China and is the most clinically advanced to baloxavir in the CENI category.

ZX-7101A is a selenium-based CENI that underwent Phase 2/3 clinical trials, and is structurally novel (Wang et al., 2025). ZX-7101A was shown to reduce the time to symptom alleviation significantly in a randomized controlled trial with one dose of the agent, with PA-I38T resistance developing in 1.8% of patients receiving treatment (Wang et al., 2025). The Phase 1–3 trial results make ZX-7101A another approved single-dose CENI that has an equivalent resistance profile to suraxavir (Wang et al., 2025; Wu et al., 2025). It has received regulatory approval in China.

### 3.4 PB1 and PB2 polymerase inhibitor

#### 3.4.1 Favipiravir (PB1 inhibitor)

The purine nucleobase analog favipiravir (T-705; 6-fluoro-3-hydroxy-2-pyrazinecarboxamide; Avigan^®^) was first developed by Toyama Chemical and authorized in Japan in 2014 for the treatment of new influenza strains resistant to current treatments (Furuta et al., 2013). Favipiravir inhibits the PB1 RdRp active site through two different mechanisms: (1) selective phosphoribosylation by cellular enzymes to the active triphosphate form, favipiravir-RTP; and (2) mutagenic incorporation into the viral RNA genome, causing error catastrophe through lethal mutagenesis (Baranovich et al., 2013; Sangawa et al., 2013).

Biochemical and structural studies have shown that favipiravir-RTP is selectively identified by viral RdRp due to the viral polymerase’s lower fidelity compared to host DNA- and RNA-directed polymerases (Jin et al., 2013). This selectivity contributes to the compound’s good therapeutic index in cell culture systems. Favipiravir-RTP mimics purine nucleoside triphosphates and is incorporated into nascent viral RNA by the viral RNA-dependent RNA polymerase, inducing predominantly C-to-U and G-to-A transition mutations through ambiguous base-pairing, leading to lethal mutagenesis of the viral genome (Jin et al., 2013; Arias et al., 2014).

The broad-spectrum activity of favipiravir is one of its most notable properties. It has shown activity *in vitro* and *in vivo* against a wide spectrum of RNA viruses other than influenza, such as Ebola, Lassa, rabies, and SARS-CoV-2, indicating that these pathogens rely on RdRp-mediated replication (Delang et al., 2018). During the 2014–2016 West African Ebola epidemic, favipiravir was tested in clinical studies using emergency use guidelines, yielding varied but clinically significant outcomes (Sissoko et al., 2016).

Favipiravir has been proven to be effective against both seasonal and pandemic strains of influenza, including highly pathogenic avian influenza (HPAI) H5N1 and the 2009 pandemic H1N1 strain (Furuta et al., 2009; Smee et al., 2009). Phase 3 clinical trials indicated non-inferior efficacy to oseltamivir in uncomplicated influenza; however, its use is still not approved for routine use in many countries due to teratogenicity concerns raised by animal research, which precludes usage in pregnant women (Hayden et al., 2022). Drug-resistant variants have been isolated *in vitro* under selection pressure (most notably K229R in PB1), but favipiravir resistance appears to entail a significant fitness cost, limiting development in clinical settings (Goldhill et al., 2018).

#### 3.4.2 Pimodivir (PB2 inhibitor)

Pimodivir (VX-787; JNJ-63623872) is a non-nucleoside inhibitor that selectively targets the PB2 subunit’s cap-binding domain, competitively limiting PB2’s interaction with the 5′ m7G cap of host pre-mRNAs (Clark et al., 2014; Byrn et al., 2015). By blocking cap recognition, pimodivir inhibits capped primers from being delivered to the PA endonuclease for cleavage, halting viral mRNA synthesis at the initiation stage upstream of PA endonuclease activity (Byrn et al., 2015).

The crystal structure of pimodivir attached to the PB2 cap-binding domain revealed that the molecule occupies the m7G binding pocket, engaging the aromatic ring of Phe404 via a stacking interaction and generating hydrogen bonds with Lys376, Asn429.4, and Glu361 (Clark et al., 2014). This binding method is extremely selective for influenza PB2 over mammalian cap-binding proteins (e.g., eIF4E), which explains the observed selectivity for influenza A viruses (Clark et al., 2014). Pimodivir does not inhibit influenza B virus replication due to structural differences in the PB2 cap-binding domain between influenza A and B viruses, which prevent effective binding of the compound and thereby limit its antiviral activity (Clark et al., 2014; Byrn et al., 2015).

In Phase 2 clinical studies, pimodivir monotherapy at 600 mg or 900 mg twice daily effectively reduced influenza virus load compared to placebo in otherwise healthy individuals with influenza A infection (Finberg et al., 2019). However, resistance evolved swiftly in monotherapy arms, with F404Y (disrupting the essential pi-stacking interaction), H357N, and L295P mutations found in a significant number of patients, emphasizing the challenges of single-agent PB2 inhibitor therapy (Trevejo et al., 2018; Finberg et al., 2019).

Combination therapy has been recognized as the most promising approach for the clinical development of pimodivir. In fact, the clinical validation for the combination of the polymerase and neuraminidase inhibitors has been obtained from the results of a Phase 2b randomized clinical trial of the combination of pimodivir and oseltamivir, which has shown not only superior antiviral efficacy but also significantly reduced risk of the emergence of drug-resistant viruses compared to oseltamivir monotherapy (Finberg et al., 2019). However, the subsequent Phase 3 BLOCKADE-2 trial evaluating pimodivir plus oseltamivir in hospitalized influenza A patients was terminated early due to lack of efficacy (Uyeki and Belser, 2025). It significantly limits the clinical development prospects of pimodivir as a combination agent in severe disease.

Other than pimodivir, onradivir (ZSP1273) is a PB2 cap-binding domain inhibitor derived by structural optimization of the pimodivir scaffold with substantially improved antiviral potency. A Phase 3 randomized, double-blind, placebo- and oseltamivir-controlled trial conducted at 68 clinical sites in China demonstrated that onradivir 600 mg once daily for five days significantly shortened time to symptom alleviation versus placebo and showed a non-inferior safety profile to oseltamivir (Yang et al., 2025). Onradivir received regulatory approval in China in May 2025 (Lee, 2025), making it the first approved PB2 inhibitor, and directly superseding the narrative that PB2 inhibitor development was halted following the discontinuation of pimodivir.

PA endonuclease inhibitors have succeeded where PB2 inhibitors initially failed, and the structural reason is clear. The PA active site offers a well-defined metal-chelating cavity with high ligand affinity. By contrast, the PB2 cap-binding pocket resembles a protein-protein interface, where a single resistance substitution (F404Y or H357N) is sufficient to abolish the critical pi-stacking interaction. Onradivir is the first approval under a clinical strategy for managing resistance in PB2 that involves scaffold optimization instead of reliance on a single drug. The lessons for pipeline development are that near-term additions to the antiviral armamentarium have a high value, in the form of the next generation of CENIs with altered contact geometry at the PA active site (e.g. suraxavir, ZX-7101A, etc.) and that the PB2 target is not a single point that can be attacked in isolation because of concerns about emergence of resistance, as the failure of pimodivir BLOCKADE-2 illustrated.

### 3.5 Emerging polymerase subunit targets (PA, PB1)

Aside from the recognized PA endonuclease and PB2 cap-binding domains, new research has found additional sites within the polymerase complex that may be susceptible to pharmacological disruption. The protein-protein interaction (PPI) interfaces of polymerase subunits, particularly the PA C-terminal/PB1 N-terminal interface and the PB1 C-terminal/PB2 N-terminal contact, have been identified as allosteric inhibitory sites (Muratore et al., 2012; Zhang et al., 2020).

The PA-PB1 interaction involves a short β-strand peptide at the PB1 N-terminus (residues 1-12) that inserts into a hydrophobic cleft on the PA C-terminal domain. In cell culture, peptide-based inhibitors generated from the PB1 N-terminal sequence demonstrated antiviral action, validating the interface’s pharmacological tractability (Ghanem et al., 2007; Wunderlich et al., 2011). Small-molecule screens targeting this site have found drugs with low-micromolar inhibitory doses and wide effectiveness against a variety of influenza A strains (Obayashi et al., 2008).

The PB1 polymerase domain is a typically ‘difficult’ therapeutic target due to structural similarities between viral and host RdRps; nonetheless, nucleoside analog prodrugs that preferentially engage the viral PB1 active site are still being discovered and optimized (Deval, 2009). Aside the endonuclease active site, the PA N-terminal region has other surfaces as well. Chemicals such as green tea-derived catechins and synthetic derivatives have been used to target the RNA-binding groove of PA-N, which is different from the metal-coordinating catalytic residues. These chemicals restrict RNA substrate access without directly chelating the active metals (Kuzuhara et al., 2009). Although most such drugs are still in early preclinical phases, they represent a mechanistically unique strategy that may give complementing or synergistic action with traditional CEN inhibitors such as baloxavir.

**Figure 6:**
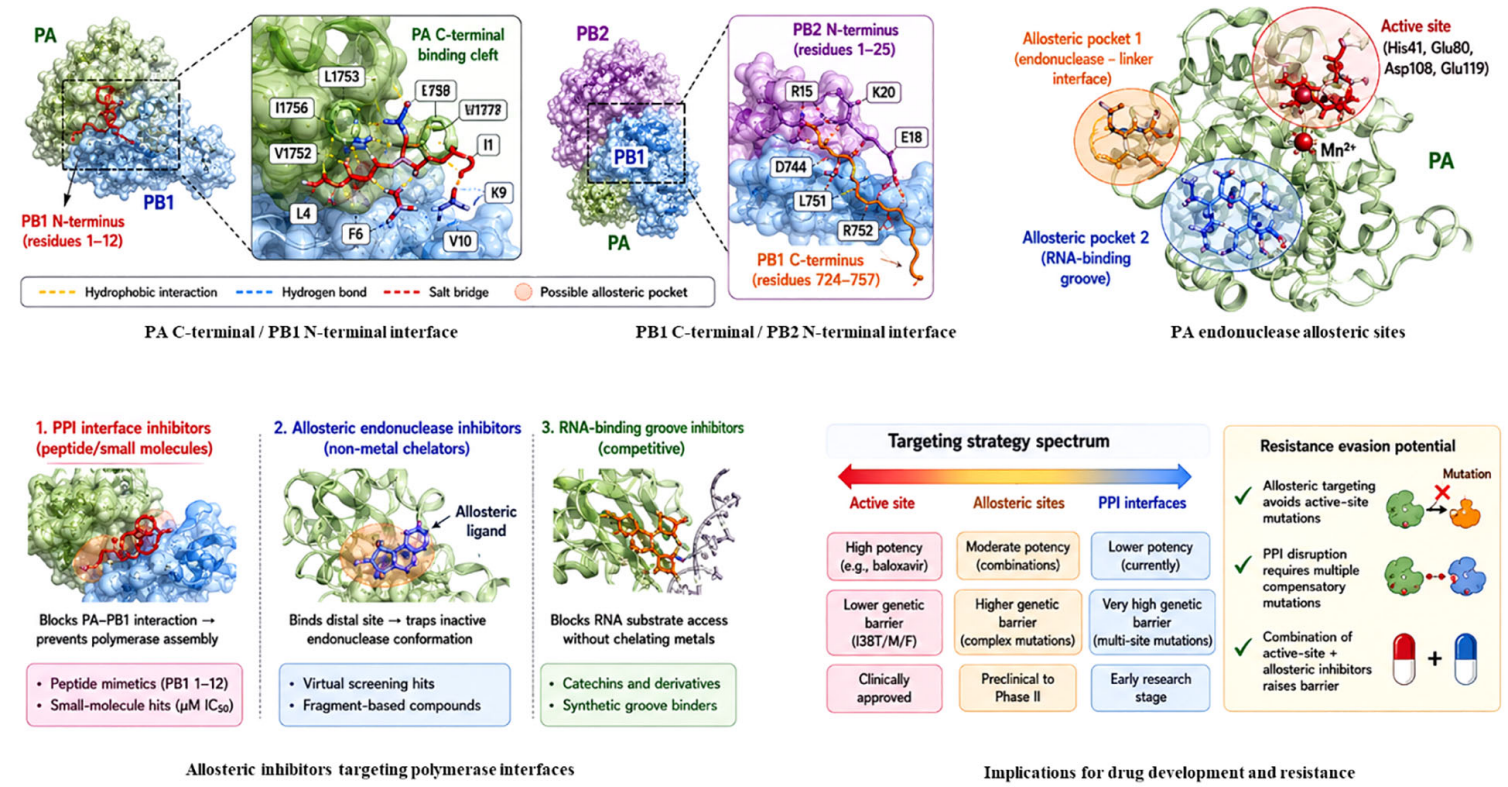
Polymerase subunit protein-protein interaction (PPI) interfaces as antiviral targets. Top: the PA-PB1 binding interface and the PB1-PB2 N-terminal interface, both stabilized by hydrophobic and electrostatic contacts (key residues labeled), alongside the PA endonuclease domain showing its active site and two pharmacologically accessible allosteric pockets. Bottom: three pharmacological strategies for disrupting polymerase function (PPI interface inhibitors, allosteric endonuclease inhibitors, and RNA-binding groove inhibitors), compared by potency, genetic barrier to resistance, and current stage of clinical development.

Allosteric inhibition of the PA endonuclease, which targets areas distal to the metal-binding active site, has emerged as another approach for overcoming I38X resistance mutations (Credille et al., 2018). Virtual screening and fragment-based drug discovery (FBDD) methods have revealed small compounds that bind to the interface of PA’s endonuclease and linker domains, potentially trapping the protein in a catalytically inept conformation (Credille et al., 2018). These findings combined suggest that the influenza polymerase complex remains a rich source of tractable antiviral targets, and that the evolving structural knowledge of the trimeric RdRp will continue to drive innovation in this therapeutic domain (**Figure 6**).

## 4 Direct-acting antiviral targets beyond polymerase

The success of neuraminidase inhibitors and, more recently, PA endonuclease inhibitors has reinforced the proof of principle for direct-acting antivirals (DAAs) for the treatment of influenza. However, the recent emergence of oseltamivir-resistant H1N1 strains with the H275Y change in the neuraminidase protein has emphasized the need to improve the diversity of the target landscape for the treatment of influenza (Dharan et al., 2009; Hurt et al., 2012). Apart from the well-characterized targets of the influenza virus, such as the neuraminidase protein and the RNA-dependent RNA polymerase (RdRp) complex, several other proteins have the potential for the development of new antiviral drugs with novel mechanisms of action and non-overlapping resistance profiles. These include the hemagglutinin protein and its fusion machinery, the nucleoprotein, which forms the core structure of the ribonucleoprotein (RNP) complex, the multifunctional non-structural protein 1, and the matrix proteins M1 and M2.

### 4.1 Hemagglutinin inhibitors

#### 4.1.1 Fusion inhibitors targeting HA2

Hemagglutinin (HA) mediates two essential steps in the influenza life cycle: receptor binding through the globular HA1 head, and membrane fusion through the HA2 stalk domain (Wilson et al., 1981). As a result of endosome acidification, hemagglutinin changes conformation irreversibly, with HA2 changing from a metastable pre-fusion conformation to a hairpin-shaped post-fusion conformation, resulting in fusion of the viral and endosome membranes, releasing the viral genome into the cytoplasm (Skehel and Wiley, 2000). The stalk of HA2 is structurally conserved across different influenza strains, thus making it a promising candidate for fusion inhibitors that remain effective despite HA1 antigenic drift (Ekiert et al., 2009; Corti et al., 2011).

Small compounds targeted to inhibit HA2 refolding usually bind to the hydrophobic pocket at the base of the HA stem, stabilizing the pre-fusion trimer and inhibiting the spring-loaded conformational transition (Kadam et al., 2017). These compounds are from various classes, including indoles, thieno[2,3-d] pyrimidines, and cyclic peptide mimetics. A study by Kadam et al. was instrumental in determining the crystal structure of a fusion inhibitor, JNJ-4796, in complex with the HA stem of H1N1, thus showing that the fusion inhibitor binds within a hydrophobic groove, forming a hydrogen bond with Asp19 of HA2 (Kadam et al., 2017). By following this lead, compounds with pan-influenza A virus activity at nanomolar concentrations in cell-based assays have been developed (Kadam et al., 2017).

Resistance profiling suggests the genetic barrier for H1 strains is higher than for H3, with mutations tending to shift the pH threshold of conformational change rather than directly disrupting the inhibitor binding site (Wu et al., 2020).

#### 4.1.2 Arbidol (umifenovir): mechanism and evidence

Umifenovir (sold under the brand name Arbidol) is an antiviral agent that has been approved for use in Russia and China for the prevention and treatment of influenza A and B (Blaising et al., 2014). Umifenovir has a unique place among antiviral agents as an HA fusion inhibitor, as crystal structures have revealed that it binds in the HA trimer interface at the stem base, making π-stacking interactions with Tyr94 and Trp21 of the HA2 helix (Kadam and Wilson, 2017). By stabilizing the neutral-pH conformation of HA, umifenovir blocks the acid-triggered refolding required for membrane fusion, therefore inhibiting viral entry at the post-attachment, pre-fusion stage (Leneva et al., 2009).

Clinical evidence of the efficacy of umifenovir has been the subject of much debate. A multicenter double-blind randomized placebo-controlled trial (the ARBITR study) discovered that umifenovir considerably reduced viral shedding and expedited symptom resolution compared to placebo, while the effect sizes were small (Kiselev et al., 2015). A meta-analysis of umifenovir and oseltamivir indicated no significant difference in time-to-symptom relief, and the two compounds had a similar safety profile (Feng et al., 2022). It is worthy of note that umifenovir is active against oseltamivir-resistant H1N1 strains of the influenza virus, as the target of umifenovir, the HA fusion machinery, is completely orthogonal to the active site of the influenza virus (Leneva et al., 2016).

Apart from the inhibition of viral fusion, umifenovir has also been seen to have effects on innate immune signaling by the induction of interferon-stimulated genes, although the role of immunomodulatory effects in the clinical efficacy of the drug is still uncertain (Blaising et al., 2014). Pharmacokinetic parameters have also been established for umifenovir, showing that it is absorbed quickly after oral administration, with Tmax at 1.5 hours, extensive protein binding (~90-99%), and being metabolized by CYP3A4 into an active sulfoxide metabolite (Deng et al., 2013). Resistance to umifenovir has been seen in HA2 gene mutations K51N, K117R, Q27N, Q42H, although clinical resistance is still very rare (Leneva et al., 2009).

Nucleoprotein (NP) and NS1 protein have also been explored as antiviral targets; however, the absence of clinical candidates with a viable resistance profile has limited their progression beyond early preclinical stages.

### 4.2 Revisiting matrix protein targeting: M2 and M1

The matrix proteins M1 and M2 arise from alternatively spliced mRNAs encoded by segment 7 of the influenza genome and play a complementary role in the life cycle of the virus (Lamb and Choppin, 1983). M2 is a homotetrameric channel for the selective permeation of protons, activated by the low pH of the endosome to acidify the interior of the virus for the dissociation of the M1 protein from the RNP complex (Pinto et al., 1992). M1, the major structural protein within the virion, lines the interior matrix of the virion and determines its shape while also regulating RNP export from the nucleus (Nayak et al., 2004). Both proteins have been the subject of therapeutic targeting with vastly different success.

The adamantane family of M2 channel inhibitors, including amantadine and rimantadine, were among the first antiviral compounds to gain approval against influenza virus and achieved their effects by occluding the M2 channel and thereby inhibiting proton conductance (Davies et al., 1964). However, the near ubiquitous presence of the S31N change within circulating influenza A strains has made these compounds essentially obsolete as clinical tools, with the World Health Organization announcing that resistance to adamantanes has risen to over 99% among current circulating strains of H3N2 and seasonal H1N1 virus (Deyde et al., 2007). This has reinvigorated interest in the development of compounds that can inhibit the S31N form of the M2 channel.

The medicinal chemistry of inhibitors that are active against the resistant S31N form of M2 has continued to progress. Spiroadamantane amine derivatives and rimantadine analogs with modified adamantane scaffolds have demonstrated partial inhibitory activity against S31N M2 in electrophysiological assays, but none has progressed to clinical development (Schnell and Chou, 2008; Wang et al., 2009; Wang et al., 2013). The primary pharmacological problem is that S31N increases the electropositivity of the pore and decreases the binding affinity of adamantane-class blockers, and no compound has so far proved to be both a potent inhibitor of S31N and to have the pharmacokinetic profile necessary to advance to clinical development.

An important exception to the clinical irrelevance of adamantanes is the *in vitro* susceptibility of current clade 2.3.4.4b H5N1 viruses to amantadine and rimantadine, in contrast to seasonal H3N2 strains where S31N resistance is universal (Deyde et al., 2007). Despite this retained susceptibility, however, adamantanes are not recommended for use in the clinical treatment of H5N1 infection because of the known tendency to rapidly develop *de novo* S31N resistance upon selective drug pressure within a single course of treatment, the lack of clinical benefit data, and regulatory recommendations that exclude this class from treating new influenza A virus infections in humans. The only recent H5N1 infection in which amantadine was used in the context of clinical practice, however, was in the critically ill British Columbia adolescent in 2024, as part of an emergency triple-combination therapy that was not designed as a standard treatment and was used before susceptibility data were available (Jassem et al., 2025).

## 5 Host-directed therapies

### 5.1 Rationale for targeting host factors

The advances in direct-acting antiviral design also highlights the importance of complementary host-directed approaches aimed at enhancing antiviral breadth and mitigating the emergence of resistance. Host-directed therapies (HDTs) are a conceptually separate paradigm: instead of targeting constantly evolving viral proteins, they modify conserved host molecules that the virus uses for replication, immune evasion, or pathological inflammation. Because of the significantly slower evolutionary rate of host genomes compared to viral genomes, resistance emergence to HDTs is inherently less likely to occur (Planz, 2022). Moreover, many of the obligate host requirements are conserved across influenza A and B subtypes, as well as other respiratory viral pathogens, thus offering the possibility of pan-respiratory activity of a single therapeutic agent (Watanabe et al., 2010). HDTs can also treat the immunopathological component of severe disease, a dimension that DAAs are not able to address since a significant proportion of morbidity and mortality in pandemic influenza is due to uncontrolled host immunopathology rather than direct viral cytopathology (Tisoncik et al., 2012).

High-throughput functional genomic studies further strengthened the scientific case for HDTs. Genome-wide RNA interference (RNAi) screens in human cells have identified hundreds of host dependence factors whose removal significantly reduces influenza replication, providing a methodical roadmap for therapeutic intervention (Hao et al., 2008; Karlas et al., 2010). Overall, HDTs are a key complement to conventional antivirals in influenza management due to their clear molecular explanation, reduced resistance barrier, and potential to reduce viral propagation and immunopathology at the same time.

### 5.2 Targeting host cell entry machinery

#### 5.2.1 Sialidase and receptor analogs

Influenza hemagglutinin (HA), the viral attachment protein, binds to α2,3- or α2,6-linked sialic acid (SA), which is presented on the glycoproteins and glycolipids on the respiratory tract epithelial cells. This interaction is essential for viral infection and is therefore considered a potential target for therapeutic intervention. To block viral infection, two approaches have been used: enzymatic depletion of SA receptors on the surface of the infected cells using exogenous sialidases and competitive inhibition using multivalent SA receptor analogs (Matrosovich et al., 2015).

DAS181, also known as fludase, is a recombinant fusion protein of a human lung sialidase and is inhaled to cleave both α2,3- and α2,6-linked SA from airway epithelial cells, removing all possible HA binding sites before viral interaction. Preclinical studies in mice and ferrets infected with both seasonal and pandemic influenza strains showed potent antiviral activity, even against oseltamivir-resistant strains, due to a mechanism of action entirely independent of viral NA activity (Malakhov et al., 2006). Clinical studies in immunocompromised patients with severe respiratory viral infections have shown a reduction in viral load and time to clinical improvement (Moss et al., 2012). The epithelial lining of the lung has a high turnover of SA groups, minimizing systemic SA depletion and providing a favorable safety profile.

Polyvalent sialylated polymers that compete with HA for receptor binding represent a second preclinical entry-inhibition strategy, though no candidate has entered clinical evaluation (Lauster et al., 2020).

### 5.3 Immunomodulatory approaches

#### 5.3.1 Cytokine storm mitigation strategies

Among the few immunomodulatory approaches in severe influenza that have been studied; corticosteroid therapy is the most studied and has shown no clinical benefits. It is also linked to higher mortality rates in observational studies and systematic reviews in severe influenza. This negative evidence establishes the benchmark for evaluating mechanistically targeted cytokine modulation strategies. Tocilizumab, a monoclonal antibody blocking the IL-6 receptor, has demonstrated reduced mortality and progression to mechanical ventilation in COVID-19-associated cytokine release syndrome, and has been proposed as a candidate therapy for influenza-associated ARDS given that IL-6 is significantly elevated in severe influenza, though direct clinical trial evidence in influenza remains limited (Abani et al., 2021). Anakinra, a recombinant IL-1 receptor antagonist, has been proposed based on IL-1β’s role in the NLRP3 inflammasome-dependent response to influenza infection. Clinical evidence from hyperinflammatory critical illness suggests potential benefit (Kyriazopoulou et al., 2021).

The agonism of sphingosine-1-phosphate (S1P) receptors is another mechanistically distinct approach. In a study by Teijaro et al., it was found that S1P1 receptor expression is predominantly found in pulmonary endothelial cells and lymphocytes, and agonizing this receptor results in reduced cytokine production and innate cell recruitment without affecting antiviral clearance (Teijaro et al., 2011). This study provided proof of concept that immunopathological and antiviral responses can be pharmacologically dissociated in murine influenza models.

Inhaled IFN-β and type III IFN-λ have shown preclinical promise by enhancing cytolytic T cell responses and providing mucosal-specific antiviral activity, respectively, but neither has Phase 3 efficacy data in influenza (Koerner et al., 2007; Wack et al., 2015).

### 5.4 Modulation of host signaling pathways

#### 5.4.1 RAF/MEK/ERK pathway inhibition

The RAF/MEK/ERK MAPK cascade, a key cell survival pathway, is activated within 30 minutes of influenza infection and is exploited by the virus to support replication. ERK activation was seen to aid the nuclear export of the viral ribonucleoprotein (vRNP) complex, which is an essential aspect of the viral lifecycle (Pleschka et al., 2001; Marjuki et al., 2006).

The host-directed mechanism was confirmed by inhibiting this pathway with MEK inhibitors, which reduced influenza replication both *in vitro* and *in vivo* without exhibiting direct antiviral efficacy against viral proteins. In lethal murine infection models, U0126, a prototypical MEK1/2 inhibitor, lowered viral titers by several logs while improving survival (Pleschka et al., 2001). The antiviral effect was maintained against oseltamivir-resistant strains, suggesting the possibility of combination therapy; MEK inhibition paired with zanamivir demonstrated synergistic efficacy in cell culture (Ludwig et al., 2004). Zapnometinib (ATR-002), a clinical-stage MEK inhibitor developed exclusively for antiviral applications, has shown significant action against numerous influenza subtypes in preclinical animals; Phase II testing in influenza patients is currently underway (Schreiber et al., 2022).

One important issue in translation with HDTs that target the RAF/MEK/ERK pathway is how suppression of this pathway will impact the immune system, since ERK signaling is also involved in T cell activation and cytokine production. In preclinical models, it appears that a transient, early-onset MEK inhibitor regimen in an acute infection does not substantially impact adaptive immunity, although we will need to optimize pharmacokinetics (PK) and pharmacodynamics (PD) characteristics to preserve immune function (Koch-Heier et al., 2024).

The PI3K/Akt/mTOR axis offers a complementary node: mTORC1 inhibition with rapamycin showed antiviral efficacy in H1N1 rodent models and was associated with improved outcomes in a retrospective cohort of critically ill patients when combined with steroids (Ehrhardt et al., 2006; Wang et al., 2014). This retrospective association should be interpreted with caution given the absence of a concurrent comparator arm and the immunosuppressive co-administration of corticosteroids, which independently confounds the attribution of any benefit to mTOR inhibition.

Targeting NF-κB via IKK inhibitors reduces cytokine production and improves survival synergistically with MEK inhibition in animal models, though no clinical candidate has been evaluated (Pinto et al., 2011).

## 6 Biologics and immunotherapy

Biologic approaches for the treatment of influenza infections focus on the utilization and modulation of the effector mechanisms of the immune system rather than the direct inhibition of viral enzymes. In contrast to antiviral agents that target viral enzymes and can be subject to the development of resistant strains by point mutations in their target enzymes, passive Immunotherapeutics and immunomodulators target multiple conserved epitopes or utilize the body’s inherent defense mechanisms that are less likely to be overcome by viral evolutionary mechanisms. This section discusses the current state of monoclonal antibody (mAb) development against three major influenza surface antigens: the hemagglutinin (HA) stalk domain, neuraminidase (NA), and matrix protein 2 ectodomain (M2e), passive immunotherapy with convalescent plasma and hyperimmune immunoglobulin, and the emerging concept of therapeutic vaccination as an adjunct to active infection management.

### 6.1 Monoclonal antibodies against influenza virus

The rapid evolution of circulating influenza A and B strains due to antigenic drift and shift has repeatedly circumvented strain-matched vaccines, making first-generation direct-acting antivirals susceptible to resistance. Monoclonal antibodies (mAbs) targeting conserved influenza epitopes provide an alternative therapeutic option that is capable of offering broad strain coverage, a long half-life that allows for single-dose administration, and effector functions mediated through the Fc region of mAbs, including antibody-dependent cellular cytotoxicity (ADCC), antibody-dependent cellular phagocytosis (ADCP), and complement-dependent cytotoxicity (CDC). Several mAb candidates targeting the HA stalk, neuraminidase active site, and M2e have progressed through clinical trials, with generally acceptable safety profiles but variable efficacy, revealing key pharmacokinetic and mechanistic barriers to successful biologic therapy for influenza (Sun et al., 2024; Mota and Moro, 2025).

#### 6.1.1 Broadly neutralizing antibodies targeting HA stalk

The influenza HA glycoprotein is composed of two structurally unique domains: the immunodominant globular head (HA1), which is responsible for the binding of the sialic acid receptor, and the membrane-proximal stalk region (HA2), which is involved in the endosomal membrane fusion event and is more conserved between influenza strains because of lower immune selective pressure (Krammer and Palese, 2013). Stalk-directed broadly neutralizing antibodies (bnAbs) typically prevent the HA1 subunit from shedding from its trimeric prefusion structure, sterically block the fusion peptide, or prevent the pH-triggered conformational rearrangement necessary for viral membrane fusion. Stalk-reactive bnAbs can in principle neutralize the whole range of currently circulating and zoonotic influenza A subtypes because the stalk epitopes are conserved among phylogenetic Group 1 subtypes (H1, H2, H5, H6, H8, H9, H11, H12, H13, H16) and Group 2 subtypes (H3, H4, H7, H10, H14, H15) (Krammer and Palese, 2013; Bangaru et al., 2018).

VH1–69 germline-encoded antibodies targeting the HA2 stalk hydrophobic groove and anchor-epitope antibodies with cross-group H1/H2/H5 reactivity represent the two best-characterized bnAb classes, both elicited by pandemic or heterosubtypic exposures rather than standard vaccination (Margine et al., 2013; Guthmiller et al., 2022).

Numerous stalk-directed bnAbs have progressed to clinical trials with usually satisfactory safety but inconsistent effectiveness results. In Phase 2 trials, MEDI8852 showed non-inferior antiviral activity to oseltamivir in outpatients but no additive benefit in combination. MHAA4549A reduced nasopharyngeal viral shedding by 97.5% in human challenge studies but failed to improve respiratory function recovery versus oseltamivir alone in 166 hospitalized patients in the CRANE Phase 2b trial, illustrating the pharmacokinetic bottleneck: systemically administered antibodies achieve only 3-4% of serum concentration at the airway mucosa, falling below inhibitory thresholds at the site of viral replication (McBride et al., 2017; Lim et al., 2020).

#### 6.1.2 Antibodies targeting neuraminidase

Neuraminidase (NA), the second major surface glycoprotein, is involved in the cleavage of terminal sialic acid residues from host glycoproteins, facilitating the release and spread of progeny virions. The NA enzymatic active site, which is characterized by eight conserved catalytic residues and eleven framework residues, is physically tractable for widely reactive antibody design because it is maintained under high functional constraint throughout all nine NA subtypes, in contrast to the hypervariable HA head (Abbadi and Mousa, 2023). Sera cross-reactive to as few as two NA subtypes (N1 and N2) offer theoretical coverage across all existing seasonal strains as well as a variety of pandemic prospects, including H5N1, H2N2, and H9N2. Anti-NA immunity is functionally analogous to anti-HA responses (Stadlbauer et al., 2019; Abbadi and Mousa, 2023).

The study by Stadlbauer et al. identified three human mAbs (including 1G01 and 1E01) derived from an H3N2-infected donor that have the ability to bind to multiple influenza A and B NAs with exceptional breadth. The study also found that the mAbs inhibit enzymatic activity by inserting a long CDR H3 loop directly into the active site. The study also showed that the mAbs have therapeutic efficacy *in vivo* as late as 72 hours post-infection in mouse models, a time frame well beyond that of inhibitors of influenza NAs alone (Stadlbauer et al., 2019). Momont et al. have also identified the pan-influenza mAb FNI9, which has the ability to inhibit all influenza A NAs of both groups 1 and 2, as well as both influenza B NAs derived from the Victoria and Yamagata lineages. The study found that FNI9 does so by molecular mimicry of the interaction between the sialic acid carboxyl group and the three conserved arginine residues (R118, R292, R371) at the catalytic center of influenza NAs. FNI9 also has the ability to inhibit oseltamivir-resistant H274Y mutant NAs and has been shown to have synergistic activity with anti-HA stalk antibodies (Momont et al., 2023).

Other than the catalytic site, conserved epitopes located on the lateral face of the NA tetramer offer further targets for antibody action that have unique profiles for antiviral resistance. The mAb CD6 targets a conserved 30 amino acid spanning epitope located on the lateral face of a NA dimer consisting of two monomers and blocking viral spread by steric inhibition rather than enzymatic inactivation (Abbadi and Mousa, 2023). The human mAb DA03E17, derived from an infected subject infected with an H1N1pdm09 strain, targets a near-active site epitope that retains the ability to bind to NA proteins that harbor key oseltamivir resistance mutations (H274Y, I222V, and S246N) and demonstrates cross-strain reactivity to all Group 1 and Group 2 influenza A and both influenza B virus lineages by structural characterization by cryo-EM (Yasuhara et al., 2022). Anti-NA mAbs can also mediate Fc-dependent effector functions that are not related to enzyme inhibition, offering a two-pronged therapeutic approach that is particularly relevant when high levels of intracellular virus are already present when treatment is initiated. To date, no anti-NA mAb has entered a pivotal clinical trial of efficacy in influenza, but the breadth of preclinical protection, the conserved nature of these critical epitopes, and the ability of these mAbs to be active even when given at later time points post-infection suggest that these agents should be pursued for clinical development (Stadlbauer et al., 2019; Abbadi and Mousa, 2023).

#### 6.1.3 Anti-M2e antibodies

The matrix protein 2 (M2) channel is found to be highly expressed on the surface of influenza A-infected cells and to a lower density on the surface of influenza A virions. The 23-amino-acid-long ectodomain of the M2 channel (M2e) is the most conserved surface sequence of influenza A viruses. The N-terminal nine amino acids of the M2e are virtually invariant among all H1N1, H3N2, and highly pathogenic avian influenza viruses such as H5N1 and H7N9. The seasonal influenza A viruses have no more than 1 to 2 amino acid differences in their M2e sequences compared to the consensus sequence (Saelens, 2019). Despite the exceptionally high degree of conservation of the M2e sequence among influenza A viruses, immunity to M2e is found to be immunorecessive following natural infection with influenza A viruses, with only about 18% of naturally infected individuals mounting detectable anti-M2e antibodies (El Bakkouri et al., 2011; Saelens, 2019).

Anti-M2e antibody protection is Fc-dependent rather than directly neutralizing: M2e-specific IgG forms immune complexes on infected cells with high-density surface M2, which engage activating Fc gamma receptors (FcgRI, FcgRIII, FcgRIV) on alveolar macrophages and NK cells, driving phagocytosis and ADCC of infected cells to limit viral dissemination. Genetic depletion studies have demonstrated that alveolar macrophages are the primary effector cell type, and that FcgR interaction is both necessary and sufficient for protection; direct neutralization of free virus contributes only marginally (El Bakkouri et al., 2011). The IgG isotype has a significant impact on this protection: IgG2a antibodies, which engage high-affinity FcgRI with greater potency than IgG1, show significantly greater reductions in lung viral titres and reduced pulmonary injury in murine challenge models than IgG1 isotype antibodies bearing identical Fab regions (Sun et al., 2023).

In a recent study, Kim et al. showed that a triple combination of non-neutralizing IgG2a mAbs targeting distinct, overlapping epitopes within the conserved M2e N-terminus provides robust prophylactic and therapeutic protection in mice challenged with a variety of influenza A strains, when administered at doses that are individually sub-protective. This triple M2e mAb combination, when administered to immunocompetent and immunodeficient hosts, did not induce viral escape mutants after serial passaging, indicating a higher resistance barrier to this therapeutic approach compared to NA inhibitors or HA head antibodies (Kim et al., 2025). In the only completed human efficacy study, the M2e-specific IgG1 mAb TCN-032, a monoclonal cocktail, has been linked to accelerated recovery when compared to placebo in a human H3N2 challenge study, although the results from the Phase 2a study design were not statistically significant (Saelens, 2019). Fc-optimized, non-neutralizing anti-M2e mAb cocktails are a promising platform for mutation-resistant, pan-influenza A therapy, according to preclinical and early clinical data. The main obstacle that still needs to be overcome is achieving adequate antibody concentrations at the respiratory mucosal surface after systemic administration (Saelens, 2019; Kim et al., 2025).

### 6.2 Passive immunotherapy and convalescent plasma

Historical meta-analyses of convalescent blood products used in the 1918 pandemic suggested possible mortality benefit, offering early proof of concept for passive immunotherapy (Luke et al., 2006). A systematic review of 32 studies across viral respiratory infections found consistent evidence for mortality reduction when convalescent plasma was given early, though all studies were low quality without contemporaneous controls (Mair-Jenkins et al., 2015).

Two landmark RCTs were published in The Lancet Respiratory Medicine in 2019, offering definitive proof of the limitations of current CP therapies for severe influenza. Two 2019 Lancet Respiratory Medicine Phase 3 RCTs; IRC005 (high- vs. low-titer plasma in severe influenza A) and FLU-IVIG (hyperimmune IV immunoglobulin vs. placebo in influenza A and B), both showed no significant improvement in clinical status at Day 7 or mortality (Beigel et al., 2019; Davey et al., 2019). A subsequent meta-analysis of five RCTs confirmed no benefit on mortality, ICU stay, or ventilation duration (Xu et al., 2020).The likely explanation is the mucosal PK barrier: circulating antibody concentrations achievable from donor plasma fall below the inhibitory threshold at the airway epithelium where viral replication occurs.

### 6.3 Therapeutic vaccines as treatment adjuncts

Therapeutic vaccination (during active infection) with vaccines to boost adaptive immunity has not been clinically proven to be effective in influenza. The theory has been tested in candidates based on NP (such as OVX836) and mRNA platforms using conserved antigens (HA stalk, M2e, NP), but all available evidence is currently only for prophylaxis. Next-generation mRNA universal influenza vaccines are covered in the Emerging Platforms section.

This mucosal delivery barrier has proven decisive in the failure of MHAA4549A, IRC005, and FLU-IVIG and remains the single most important obstacle to biologic therapy for influenza. The next most promising candidate is the anti-NA monoclonal antibodies, which target the same highly conserved active site that is targeted by small-molecule NAIs, and which are able to inhibit later times, but not enzymatically. To realize this potential, however, a change from intravenous systematic delivery to intranasal and inhaled delivery formats that can achieve inhibitory concentration directly at the mucosal site of infection is required.

## 7 Drug repurposing: the fast-track approach to antiviral expansion

Several computational repurposing methods, such as matching gene expression patterns (e.g., Connectivity Map (CMap/LINCS L1000)), scoring drug-disease connections (e.g., graph neural network (GNN)), and candidate identification with the help of a large language model (LLM), have played a major role in identifying the antiviral candidates discussed in the following subsections. Currently, however, the biggest hurdle for all computationally nominated influenza repurposing candidates is rigorous prospective clinical validation.

### 7.1 Statins: immunomodulatory antiviral evidence and clinical trials

Statins, which belong to the class of 3-hydroxy-3-methylglutaryl coenzyme A reductase inhibitors, are the most frequently used lipid-lowering drugs worldwide and are known to exhibit immunomodulatory and anti-inflammatory effects that go far beyond their main target of inhibiting the mevalonate pathway. Statins reduce pro-inflammatory cytokine transcription through NF-κB inhibition, inhibit MHC class II antigen expression on antigen-presenting cells, downregulate leukocyte-endothelial adhesion, suppress LDL cholesterol biosynthesis, and increase heme oxygenase-1, an enzyme with cytoprotective and anti-inflammatory qualities (Fedson, 2013). These pleiotropic effects formed the hypothesis, first systematically proposed by Fedson in 2006, that statins might mitigate the abnormal immunopathological host response to severe influenza infections, which is mediated by cytokines and results in lethal damage to the alveoli and contributes to a large proportion of influenza-related mortalities, without affecting viral clearance (Fedson, 2006).

The evidence for a preventive statin effect in influenza has been strong, albeit methodologically diverse. Statin use was associated to modest but statistically significant decreases in pneumonia hospitalization (OR 0.92; 95% CI 0.89-0.95), 30-day pneumonia mortality (OR 0.84; 95% CI 0.77-0.91), and all-cause mortality (OR 0.83; 95% CI 0.79-0.87), according to a population-based propensity score-matched cohort study of 2,240,638 statin classes (Kwong et al., 2009). The hazard ratio for 30-day mortality among statin users was 0.41 (95% CI 0.25-0.68) in the 2007–2008 season, but it was attenuated and non-significant (HR 0.77; 95% CI 0.43-1.36) in the 2009 pandemic H1N1 season, indicating potential heterogeneity of effect by viral subtype or pandemic versus seasonal context (Laidler et al., 2015). A 2022 systematic review confirmed a statistically significant reduction in influenza-associated mortality among statin users, with no evidence of impaired vaccine-induced immunity (Vahedian-Azimi et al., 2022).

Despite these observational signals, the primary obstacle for statins as influenza treatments is the lack of a randomized controlled trial with clinical outcomes in influenza. Negative RCT evidence from adjacent contexts, such as the JAMA 2013 trial showing no statin benefit on 28-day mortality in ventilator-associated pneumonia and the NHLBI ARDS Network rosuvastatin trial showing no improvement in sepsis-associated ARDS outcomes, lowered enthusiasm for statin repurposing in acute respiratory infections in general. Propensity matching can lessen but not completely eliminate the “healthy user” bias present in statin prescription patterns, where chronic statin users are consistently healthier and more adherent to preventive care than non-users. This bias may contribute to the observational benefit in influenza. An RCT on an influenza-specific statin with an immunopathological primary endpoint such as AUC on inflammatory markers or time to normalization of oxygenation saturation rather than mortality alone is an unmet research priority (Fedson, 2006; Fedson, 2013; Vahedian-Azimi et al., 2022).

### 7.2 Nitazoxanide: mechanisms beyond antiparasitic activity

The US FDA approved nitazoxanide (NTZ; 2-acetyloxy-N-(5-nitro-2-thiazolyl)benzamide) is a thiazolide-class broad-spectrum antiparasitic drug for the treatment of Giardia lamblia and Cryptosporidium parvum infections. It was later found to have strong activity against a wide range of RNA and DNA viruses. Its antiviral mechanism against influenza viruses is different from its antiparasitic mechanism. The antiparasitic mechanism involves the inhibition of the enzyme involved in anaerobic electron transfer; this enzyme is the pyruvate ferredoxin/flavodoxin oxidoreductase enzyme. On the other hand, the anti-influenza mechanism involves the interference with the post-translational processing of the hemagglutinin glycoprotein (Rossignol, 2014).

NTZ and its active systemic metabolite tizoxanide (TIZ) specifically inhibit the transport of newly synthesized HA from the endoplasmic reticulum (ER) to the Golgi apparatus, where terminal glycosylation occurs, which is required for proper HA folding, trimerization, and surface transport. The final assembly stage of the influenza replication cycle is disrupted by this blockage, which causes underglycosylated HA to accumulate in the ER and prevent it from being incorporated into progeny virions at the plasma membrane. This mechanism is effective against neuraminidase inhibitor-resistant and adamantane-resistant strains because it targets a host-dependent glycosylation pathway rather than a viral protein directly. Further, it has a high intrinsic barrier to resistance development, as demonstrated by serial passage studies that failed to select NTZ-resistant influenza variants even after extended passage (Rossignol, 2014; Tilmanis et al., 2020).

NTZ enhances innate immune signaling to produce secondary immunomodulatory antiviral effects along with the HA glycosylation pathway. NTZ activates protein kinase R (PKR) and phosphorylates eIF2α, a component of the integrated stress response pathway that restricts cap-dependent translation, which preferentially translates influenza mRNA over cellular transcripts during productive infection. Following NTZ treatment, *in vitro* studies in influenza A-infected MDCK cells have shown upregulation of PKR (100–190% increase), phospho-eIF2α (5–40% increase), and LC3-II (20–60% increase). These findings were noted in the context of characterizing the Keap1-Nrf2 antioxidant pathway rather than as primary mechanistic endpoints. While the LC3-II rise indicates increased autophagic flux that may aid in the removal of viral components, the PKR and phospho-eIF2α elevations are consistent with integrated stress response activation. These findings collectively suggest that NTZ probably interacts with several host regulatory processes in addition to its main impact on HA glycosylation processing, even if it is still uncertain how much each pathway contributes to antiviral activity *in vivo (*Huang et al., 2023).

The pivotal phase 2b/3 study (NCT01227421) included adults and adolescents with acute uncomplicated influenza and randomized them to NTZ 600 mg twice daily for five days or placebo. When compared to a placebo, NTZ treatment dramatically decreased the median time to alleviate all symptoms and a clinically significant reduction in the duration of symptoms. Across all groups, diarrhea was the most commonly reported adverse event. Preclinical combination studies provided mechanistic rationale for combination regimens by showing that NTZ + oseltamivir had higher antiviral activity than either monotherapy in preventing H1N1 infection in murine models (Haffizulla et al., 2014).

However, a later phase 2 trial (NCT02057757) of NTZ in patients hospitalized with severe acute influenza, particularly those with influenza-associated pneumonia or acute respiratory failure, failed to show a therapeutic advantage over placebo. In contrast to the positive signal in simple outpatient influenza, this negative result in severe disease reflects a pattern observed with other antivirals and host-directed agents: treatments that target early replication events are most effective in the ambulatory, early-disease setting and offer little additional benefit once the inflammatory cascade causing severe outcomes has been established. Therefore, the appropriate clinical aim for NTZ is uncomplicated early influenza, possibly in combination with oseltamivir, rather than severe hospitalized disease (Haffizulla et al., 2014; Rossignol, 2014; Tilmanis et al., 2020).

### 7.3 Diltiazem and cardiovascular drugs with antiviral properties

Diltiazem, an FDA-approved non-dihydropyridine calcium channel blocker used clinically since 1982 for arrhythmias, angina, and hypertension, inhibits L-type voltage-gated calcium channels (VGCCs). Converging lines of evidence from cell biology demonstrated that intracellular calcium dynamics play a crucial regulatory role at several stages of the influenza replication cycle, including viral endosomal entry, uncoating, nuclear export of viral ribonucleoprotein (vRNP) complexes, and plasma membrane budding. This finding provided an empirical basis for examining calcium channel blockers in this context, even though antiviral activity against influenza was not expected from this mechanism (Fujioka et al., 2013).

Diltiazem was first shown in 2019 to have potent antiviral properties against influenza A viruses in cell culture and reconstructed human airway epithelia (HAE) models, with 50% inhibitory concentrations in the low-to-mid micromolar range attainable at plasma concentration (Pizzorno et al., 2019). What is particularly interesting is that the mechanism of the drug is not related to VGCC inhibition. In fact, transcriptome analysis of diltiazem-treated airway epithelial cells showed the induction of an interferon-stimulated gene (ISG) expression profile mediated through the type III IFN signaling pathway, placing diltiazem among innate immune response amplifiers that prime airway epithelial cells into a pre-activated antiviral state, rather than among direct-acting antivirals.

A 2025 follow-up study from the same group found that the diltiazem-baloxavir combination outperformed each monotherapy against both wild-type influenza A and the PA I38T baloxavir-resistant strain in cell culture and HAE models. This orthogonal mechanism, diltiazem priming an antiviral transcriptional state while baloxavir directly inhibits viral RNA production, suggests diltiazem combination regimens could address the clinically significant issue of baloxavir-resistance emergence, specifically in immunocompromised patients, who shed virus for extended periods and serve as the primary reservoir for resistant variant spread (Padey et al., 2025).

Verapamil, another non-dihydropyridine calcium channel antagonist, similarly inhibits influenza viral entry and post-entry trafficking through disruption of calcium-dependent endosomal maturation. Numerous epidemiological studies have linked the broader class of voltage-gated calcium channel blockers, such as amlodipine and nifedipine, to lower mortality from respiratory virus infections; however, prospective trials have not supported these findings. The main obstacle to translational progress is the lack of published clinical trial data for diltiazem or other calcium channel blockers specifically in influenza. Diltiazem is a great option for a well-powered investigator-initiated clinical trial in conjunction with approved antivirals due to its favorable safety profile following forty years of cardiovascular use, its affordable generic availability, and its oral bioavailability compatible with outpatient administration (Pizzorno et al., 2019; Padey et al., 2025).

### 7.4 Metformin and AMPK activation as an antiviral strategy

Metformin (dimethylbiguanide) suppresses mitochondrial Complex I of the respiratory chain. This lowers the intrahepatic ATP/AMP ratio and activates AMP-activated protein kinase (AMPK). AMPK is considered a master energy sensor in cells, which, upon activation, inhibits anabolic processes while activating catabolic processes to restore energy balance. The antiviral importance of AMPK activation is related to the critical role that host cell metabolism plays in facilitating productive influenza replication, such as RNA synthesis, translation, lipid membrane remodeling for viral envelope biogenesis, and vRNP export, all of which require energy reserves that can be limited by AMPK-mediated metabolic suppression. Metformin, an AMPK activator, reduces influenza A virus replication in a dose-dependent manner, which is related to the suppression of mTOR complex 1 (mTORC1) activity, a master regulator of cap-dependent translation required for efficient production of viral proteins. AMPK activation inhibits NF-κB transcriptional activity through AMPK-dependent and -independent mechanisms, which may help reduce the overwhelming inflammatory response that causes lung damage during influenza infection (Halabitska et al., 2024).

Metformin also has immunomodulatory effects relevant to severe influenza, inhibiting Th17 differentiation, promoting Treg polarization, and reducing macrophage TNF-α and IL-6 output, effects particularly relevant in elderly patients where immunosenescence and metabolic dysregulation converge to drive excess influenza severity (Martin et al., 2023).

Metformin’s mTORC1 inhibition has been shown to reduce type I interferon responses, which should be taken into account when assessing its antiviral potential. A 2020 study found that metformin suppressed IFN-α expression in human PBMCs stimulated with influenza virus via mTORC1 pathway suppression. Additionally, metformin treatment in diabetic patients was linked to impaired hemagglutination-inhibition antibody persistence after seasonal influenza vaccination. This study has important implications for the treatment of diabetic patients with the influenza vaccine and the potential of metformin as an immunomodulator in the treatment of the flu. Before metformin can be suggested as an adjuvant influenza treatment outside of the diabetic context, prospective clinical trials with immunological endpoints are necessary. This dual effect - suppressing detrimental inflammatory amplification on the one hand, while possibly blunting some beneficial interferon responses on the other - highlights that the net immunological effect of metformin in influenza is context-dependent (Saenwongsa et al., 2020). The COVID-OUT trial demonstrating viral load reduction with early outpatient metformin in SARS-CoV-2 provides a methodological template for an influenza-specific repurposing trial (Halabitska et al., 2024).

The drug repurposing evidence base for influenza reflects a common pattern, which is that drugs that have a specific antiviral mechanism, such as targeting a host process (NTZ: HA glycosylation, DAS181: depletion of sialic acid receptors, diltiazem: upregulation of interferon pathway) induce Phase 2 data and fail to reach Phase 3. The major challenges are not scientific, but logistical: influenza infection is seasonal, with a short window of time for enrollment in traditional trial formats. The platform trial infrastructure that can be built to allow for the rapid enrollment of repurposed agents was shown as a result of the COVID-19 pandemic. We can use a comparable adaptive trial design for the two most promising combinations of influenza repurposing drugs, diltiazem and baloxavir, and NTZ and oseltamivir, to address the current evidence gap within one to two influenza seasons.

## 8 Antiviral resistance across drug classes

The ability of the virus to produce resistance mutations under drug selection pressure limits the therapeutic efficacy of all currently approved influenza antivirals to varied degrees. Influenza A and B viruses replicate using RNA-dependent RNA polymerases that are inherently error-prone and lack proofreading activity. This results in mutant spectra with estimated error rates of roughly 10^-^ (Dapat et al., 2025) substitutions per nucleotide per replication cycle (Pauly et al., 2017). This rate ensures that nearly every single-nucleotide mutant of the viral genome exists within a sufficient viral population at any given time. When antiviral pressure is applied, this mutational plasticity and the high viral titers attained in the respiratory tract during acute infection generate favorable conditions for the selection of drug-resistant variants. All currently approved classes of anti-influenza drugs, including adamantane M2 ion channel blockers, neuraminidase inhibitors (NAIs), and the CENI baloxavir marboxil are susceptible to resistance through single or double amino acid substitutions in their respective targets, demonstrating that a low genetic barrier to resistance is a shared vulnerability of the existing antiviral arsenal rather than a class-specific liability (Hussain et al., 2017) (**Table 2**).

**Table 2:** Clinically relevant antiviral resistance mutations.

Drug (class)	Mutation (gene; subtype)	Protein location	Molecular mechanism of resistance	IC_50_ fold change	Fitness cost & compensatory mutations	Cross- resistance profile	Community transmission evidence	Clinical management implication
Neuraminidase inhibitors								
Oseltamivir (NAI)	H275Y (NA; A/H1N1)	150-loop adjacent NA active site	Tyrosine residue causes steric displacement of oseltamivir carboxylate; does not affect smaller zanamivir scaffold	>300× vs. oseltamivir; >300× vs. peramivir	Significant fitness cost WITHOUT compensatory mutations. Permissive mutations restore fitness: D344N, V241I, N369K, R222Q. Y276F increases NA activity 70%	High-level resistance to oseltamivir AND peramivir. Full susceptibility to zanamivir and laninamivir retained	Documented (2007–2009 H1N1; Australia cluster 2012)	Switch to zanamivir or laninamivir. Use baloxavir (active vs. H275Y). Combination therapy recommended in immunocompromised patients
Oseltamivir (NAI)	R292K (NA; A/H3N2 and H7N9)	NA catalytic framework residue (conserved)	Lysine disrupts sialic acid carboxylate interaction required for all NAI binding; broad active-site distortion	>1000× vs. oseltamivir; >30× vs. zanamivir	High fitness cost in H3N2; notably LOWER fitness cost in H7N9 avian-adapted NA background. Avian H7N9: R294K emerges in ~30% of oseltamivir-treated patients	Broadest NAI cross-resistance of any single mutation; reduced inhibition across all 4 licensed NAIs. Does NOT affect baloxavir	Not well established in seasonal strains	Baloxavir remains active. Dual-mechanism combination strongly advised in H7N9 infections. Report to national surveillance
Cap-dependent endonuclease inhibitor								
Baloxavir Marboxil (CENI)	PA-I38T (PA; A/H1N1 and A/H3N2)	PA endonuclease hydrophobic pocket (I38)	Disrupts van der Waals interactions between baloxavir acid’s naphthyridine ring and hydrophobic I38 residue in PA active site	11–22× (H1N1) 41–57× (H3N2) 4–17× (Flu B)	Partial fitness cost (H3N2 I38T: modest; H1N1 I38T: larger). Compensatory mutations already circulating as of 2018–2019 season (Omoto et al., 2018). A(H3N2) I38T transmits between ferrets comparably to WT	No cross-resistance with NAIs. I38T viruses fully susceptible to oseltamivir, zanamivir	Documented (Japan H3N2 Feb 2019; epidemiologically unrelated cases)	Combine with oseltamivir to prevent emergence (eliminates I38X in murine lung-to-lung passage). Intensified global surveillance for I38X with permissive mutations via GISAID (Global Initiative on Sharing All Influenza Data)
Baloxavir Marboxil (CENI)	PA-I38F/I38M (PA; A/H1N1 and A/H3N2)	PA endonuclease active site periphery	Same hydrophobic pocket disruption as I38T but with larger amino acid substitutions; I38M somewhat less common	Variable; generally similar to I38T range	I38F and I38M have similar or slightly greater fitness cost than I38T in cell culture competition experiments	No cross-resistance with NAIs	Possible (observed in treated patients, community origin not confirmed)	Same management as PA-I38T. Note higher resistance rates in pediatric patients (up to 23.4% in <12 yrs in some studies)
PB2 inhibitor (investigational)								
Pimodivir (PB2 CBD inhibitor)	F404Y, H357N, L295P (PB2; A viruses)	PB2 cap-binding domain (Phe404 pi-stacking site)	F404Y directly disrupts the essential pi-stacking interaction between pimodivir and PB2-Phe404 aromatic ring; H357N and L295P affect adjacent binding contacts	F404Y: >100× H357N: ~30× L295P: ~30× vs. pimodivir	Fitness cost not fully characterized; emerge rapidly under monotherapy pressure in 7–10% of treated patients	No cross-resistance with NAIs or baloxavir (different target)	Not established (drug not approved; Phase 3 discontinued)	Combination with oseltamivir prevents emergence (TOPAZ trial pimodivir+OTV arm showed no resistance). PB2 resistance monitoring integrated in clinical trial protocols
M2 ion channel blockers (adamantanes - clinically obsolete)								
Amantadine/Rimantadine	S31N (M2; A/H3N2 and A/H1N1)	M2 transmembrane pore residue 31	Asparagine at position 31 creates steric clash with adamantane scaffold, preventing channel occlusion; allosterically alters pore geometry	Complete loss of inhibition (functionally infinite)	No fitness cost; S31N viruses replicate and transmit identically to wild-type. *De novo* acquisition under drug pressure occurs within single treatment course	Cross-resistance across all adamantane class compounds. Does NOT affect NAIs or baloxavir	Universal (>99% H3N2 and seasonal H1N1 strains)	Adamantanes should NOT be used as monotherapy or empirical therapy for any circulating influenza A strain. Exception: clade 2.3.4.4b H5N1 retains susceptibility *in vitro*, but clinical value unestablished and rapid *de novo* resistance expected under pressure

### 8.1 Neuraminidase mutations (e.g., H275Y)

The H275Y substitution (N1 numbering; equivalent to H274Y in N2 numbering) is the defining resistance mutation giving high-level oseltamivir resistance in influenza A(H1N1) viruses. The mutation explains the selective resistance profile: H275Y confers high-level resistance to oseltamivir (>300-fold increase in IC^50)^ and peramivir while maintaining full susceptibility to zanamivir and laninamivir (Hussain et al., 2017). It does this by introducing a tyrosine residue at a position adjacent to the 150-cavity of the NA active site, which causes steric displacement of the carboxylate group of oseltamivir but not the smaller compounds zanamivir or laninamivir. While overall resistance rates remained low (0.09%, 0.12%, and 0.23% of tested isolates, respectively), the WHO global influenza antiviral resistance surveillance network confirmed that H275Y in A(H1N1)pdm09 viruses remained the most frequently detected substitution causing highly reduced inhibition by oseltamivir and peramivir during the 2020–2021, 2021–2022, and 2022–2023 surveillance periods (Hussain et al., 2017).

The epidemiological history of H275Y demonstrates how resistance mutations can progress from clinically insignificant to epidemiologically significant through the gradual accrual of permissive compensating mutations. Before 2007, H275Y in seasonal A(H1N1) viruses carried a clear replicative fitness cost. In ferret and guinea pig models, recombinant viruses carrying H275Y showed prolonged eclipse phases (6.6 to 9.1 hours), reduced burst size (7-fold fewer virions per cell), and attenuated transmissibility, limiting resistant variants to occasional treatment-emergent cases with no sustained community transmission (Brookes et al., 2011). The D344N permissive mutation emerged in the NA of circulating A/Brisbane/59/2007-like H1N1 strains during the 2007–2008 season, significantly restoring NA enzymatic activity and allowing H275Y viruses to replicate and spread without any noticeable fitness penalty. This led to the global spread of oseltamivir-resistant H1N1 and nearly 100% resistance prevalence in a number of countries in 2008–2009 (Pizzorno et al., 2011).

The pandemic A(H1N1)pdm09 virus emerged in 2009, with a genetically different NA that was not pre-adapted for H275Y fitness, thus removing the resistant seasonal lineage from circulation and lowering global H275Y prevalence to less than 1% (Hurt et al., 2009; Pizzorno et al., 2011). A crucial proof-of-principle, however, was demonstrated by the 2007–2009 experience: H275Y can reach full transmissibility when supported by suitable permissive genetic backgrounds. Several other permissive mutations that can restore H275Y fitness in A(H1N1)pdm09 viruses have been found in community clusters of oseltamivir-resistant A(H1N1)pdm09 in immunocompromised patients (Abed et al., 2014), including V241I, N369K, N386K, and N200S. Additionally, computational prediction techniques have found additional candidate permissive substitutions (S95N, S286G, and S299A) that could help a fit H275Y A(H1N1)pdm09 variant under prolonged oseltamivir pressure (Farrukee et al., 2022).

The main oseltamivir resistance mutations in A(H3N2) viruses are found at NA residues E119, R292, and N294 R292K confers the broadest cross-NAI resistance, including zanamivir (Hussain et al., 2025). Due in part to the avian-adapted NA of H7N9 tolerating the R294K substitution with minimal fitness cost in the mammalian respiratory tract, the R294K substitution in A(H7N9) viruses has been of particular concern. It offers reduced inhibition across all four licensed NAIs and emerges under oseltamivir therapy in approximately 30% of H7N9-infected patients treated with oseltamivir (Hu et al., 2013; Hussain et al., 2025). Multi-NAI resistance has been described in immunocompromised individuals taking long-term NAI prophylaxis, in which successive selection of NA mutations can produce viruses with reduced susceptibility to all available NAIs (Hussain et al., 2025).

### 8.2 PA-I38 and polymerase resistance

The first approved CENI, baloxavir marboxil (Xofluza), inhibits the PA subunit of the influenza RNA-dependent RNA polymerase by occupying the PA endonuclease’s two-metal-ion active site, inhibiting the cap-snatching step essential to commence viral mRNA synthesis. Substitutions at residue I38 of the PA protein, most frequently PA-I38T (the prevalent form) and less frequently PA-I38F or PA-I38M, are the main cause of treatment-emergent resistance to baloxavir (Hayden et al., 2018; Takashita, 2021). Due to structural differences between type A and type B PA endonuclease active sites, these substitutions decrease van der Waals contacts between baloxavir acid and the PA endonuclease active site, resulting in 11- to 57-fold reduced susceptibility *in vitro* for representative influenza A viruses and 4- to 17-fold reduced susceptibility for influenza B viruses (Takashita, 2021).

The pivotal CAPSTONE-1 phase 3 trial demonstrated the clinical importance of PA-I38 resistance emergence, with PA-I38T or PA-I38M substitutions occurring in 2.2% of A(H1N1)pdm09-infected recipients and 9.7% of 370 A(H3N2)-infected baloxavir recipients (Hayden et al., 2018). In contrast to baloxavir recipients in whom no resistance mutation was found, patients in whom these variants appeared showed extended viral shedding, rebound in virus titers, and delayed symptom relief. In a later pediatric trial, the resistance rate was significantly higher (23.4%) among children under the age of twelve who were infected with A(H3N2) (Takashita et al., 2020). This population is known for having higher viral burdens, longer shedding times, and, as a result, more selection pressure for resistance variant selection during antiviral therapy. PA-I38 substitutions have also been detected in baloxavir-naive patients. The first confirmed community transmission of PA-I38T A(H3N2) variants was documented in Japan during the 2018–2019 season in two epidemiologically unrelated hospitalized children, only one of whom had received baloxavir, demonstrating that human-to-human transmission of baloxavir-resistant variants is possible (Takashita et al., 2019).

*In vitro* passaging experiments show that PA-I38T selection needs just six to nine passages of A/WSN/33(H1N1) in the presence of baloxavir, with the I38T substitution resulting in a 22- to 41-fold reduction in susceptibility. In competition experiments against wild-type virus in cell culture, PA-I38T viruses carrying the resistance substitution alone show a partial replication fitness cost (Lee et al., 2021). After a single passage, 50:50 mixtures evolve toward 70:30 (WT:mutant) in A(H1N1)pdm09 backgrounds and 88:12 in A(H3N2) backgrounds, which is consistent with a modest intrinsic cost of the substitution. The predicted number of transmission events (4.8, assuming equal transmissibility of wild-type and PA-I38X viruses) was significantly higher than the observed number (0), but the BLOCKSTONE, a Phase 3 randomized household transmission study of baloxavir, did not find any confirmed transmission events of PA-I38X variants from treated index patients to untreated household contacts during the study observation period (Harding et al., 2023). This finding, combined with competitive mixture ferret transmission data, suggests that current PA-I38T variants have a residual fitness deficit that limits but does not prevent community-level spread, with the risk of widespread transmission dependent on the future acquisition of compensatory permissive mutations similar to those seen in the H275Y H1N1 resistance epidemic (Imai et al., 2020).

### 8.3 PB2-associated escape variants

The PB2 subunit of the influenza RNA-dependent RNA polymerase recognizes and binds the 7-methylguanosine (m^7^GTP) cap structure of host pre-mRNA through its cap-binding domain (CBD), which is a crucial step in cap snatching. Pimodivir (VX-787), an experimental PB2 CBD inhibitor that advanced to phase 3 clinical evaluation, inhibits the cap-binding activity with nanomolar potency against influenza. Due to structural variations in the PB2 CBD between type A and type B polymerases, A viruses of all studied subtypes, including pandemic-threat H5N1, H7N9, H5N6, and zoonotic H4N2, lack action against influenza B viruses (Patel et al., 2021). According to clinical research, viruses with PB2 mutations linked to decreased pimodivir susceptibility were shed by 7-10% of pimodivir-treated patients. These substitutions occurred at nine residues spread throughout the mid, cap-binding, and RNA-binding linker regions of PB2 (Finberg et al., 2019).

Less than 0.03% of 11,934 seasonal virus sequences and 0.2% of non-seasonal zoonotic viruses were found to have PB2 substitutions linked to decreased pimodivir susceptibility among seasonal circulating viruses in US surveillance data from 2015–2020. This indicates that baseline population-level resistance to pimodivir is still uncommon in the absence of drug pressure. A rare avian H4N2 isolate with PB2-H357N showed more than 100-fold reduced susceptibility to pimodivir, and human seasonal viruses with PB2 substitutions S324C, S324R, or N510K showed 27- to 317-fold reduced susceptibility. These findings show that both pre-existing natural variation and *de novo* selection under drug pressure can produce pimodivir-resistant genotypes across influenza A diversity (Patel et al., 2021).

In contrast to pimodivir resistance, PB2 residue 627, which contains lysine (627K) in human-adapted strains and glutamic acid (627E) in avian viruses, is not a site of conventional antiviral resistance but rather a crucial determinant of host range, polymerase activity in mammalian cells, and virulence in mammalian hosts. The E627K substitution increases replication efficiency in the cooler upper respiratory tract (33 °C) (Guo et al., 2025), increases PB2 polymerase activity in mammalian cells by about 40 times through improved interaction with the host factor ANP32A, and is positively correlated with fatal outcomes in H7N9-infected patients (Liu et al., 2020). PB2-627V, a recently described variant that has emerged in poultry since the 2010s across H9N2, H7N9, H3N8, and 2.3.4.4b H5N1 subtypes. This variant is of particular concern to public health, due to the dual-characteristics of both avian-type 627E (which permits efficient replication in avian hosts and chickens) and human-type 627K (which supports mammalian replication via utilization of both avian- and human-origin ANP32A proteins) (Guo et al., 2025).

By improving PB2’s nuclear import through better binding to mammalian importin-α1, the PB2-D701N substitution offers a second mammalian adaptation pathway that is independent of E627K. It has also been shown to functionally replace 627K in supporting the mammalian transmission of avian H5N1 and H7N7 viruses (Steel et al., 2009). The emergence of multiple PB2 mutations capable of independently facilitating mammalian adaptation, including E627K, D701N, Q591K, and K526R, all of which have been detected in human zoonotic infections with avian viruses, demonstrates that the barrier to avian-to-human polymerase adaptation is a genetic landscape with multiple alternative pathways rather than a single mutational bottleneck (Steel et al., 2009; Guo et al., 2025).

### 8.4 Fitness costs and compensatory mutations

A key factor in determining whether a drug-resistance mutation is an epidemiological threat or a transient, clinically manageable treatment-emergent event is its impact on viral fitness: the resistant variant’s ability to replicate, shed, and transmit relative to the wild-type susceptible virus in the absence of ongoing drug pressure. As the 2007–2009 H275Y H1N1 epidemic showed, resistance mutations with low or no fitness costs can spread and possibly displace susceptible strains globally; and those with high fitness costs are restricted to treated hosts and do not spread effectively in the community (Brookes et al., 2011; Pizzorno et al., 2011).

The fitness ramifications of resistance mutations are decided at the molecular level by whether the substitution structurally alters protein function outside of the targeted drug-binding site. By modifying the geometry of the 150-cavity in the NA active site, the substitution for H275Y decreases NA sialidase enzymatic activity and surface expression. This disruption imposes a replication fitness cost proportionate to the degree of NA activity reduction in the affected genetic background (Brookes et al., 2011; Abed et al., 2014). Permissive compensatory mutations that rescue H275Y fitness act by restoring NA surface expression and catalytic efficiency through alternative structural rearrangements: Y276F, immediately adjacent to H275Y, was identified as a hyperactive mutation that increases NA activity by approximately 70% and selectively rescues the NA surface expression and enzymatic activity reduced by H275Y (Jiang et al., 2022); the T289M mutation in A(H1N1)pdm09 increases NA affinity 2.2-fold and Vmax 3.8-fold relative to the H275Y single mutant; and V241I, N369K, and D344N have each been characterized in clinical isolates from community clusters of oseltamivir-resistant A(H1N1)pdm09 (Butler et al., 2014).

The fitness cost of baloxavir resistance in PA-I38T is lower and depends on the subtype. In cell culture competition assays using A(H3N2) PA-I38T viruses, the mutant drops from 50% to about 12% of the population in a single passage in the absence of pharmacological pressure, suggesting a quantifiable but manageable competitive disadvantage. I38T has a higher fitness cost in A(H1N1)pdm09 conditions, where the mutant drops to about 30% of the mixture population. Studies on ferret transmission confirm that A(H1N1)pdm09 PA-I38T viruses have lower between-host fitness than A(H3N2) PA-I38T viruses (Lee et al., 2021). Imai et al. later showed that modern A(H1N1)pdm09 and A(H3N2) viruses carrying PA-I38T, isolated from baloxavir-treated patients during the 2018–2019 season, had already acquired compensatory mutations restoring replicative fitness comparable to wild-type in hamsters and efficiently transmitted between ferrets by respiratory droplets. This finding has important surveillance implications, suggesting that fit PA-I38T variants are already in circulation rather than being hypothetical (Imai et al., 2020).

Both the H275Y and PA-I38T resistance landscapes have been subjected to computational techniques for prospective prediction of permissive mutations. In order to provide a surveillance-prioritization framework, machine learning methods trained on experimental fitness data can identify NA substitutions with a high probability of compensating H275Y-associated fitness costs. If these computationally predicted permissive mutations start to circulate at detectable frequencies in global GISAID influenza sequence datasets, they should initiate intensified phenotypic fitness and transmissibility assessments prior to any observed resistance outbreak (Farrukee et al., 2022). The more restricted natural sequence diversity available for training makes it difficult to apply equivalent prospective computational analysis to PA-I38T; however, the analogy to the H275Y permissive mutation evolutionary pathway offers a conceptual road map that should inspire preemptive surveillance (Takashita, 2021).

It is easy to see why these mutations followed different epidemiological pathways when directly compared with each other against genetic barrier and transmission potential by drug class (**Table 2**). The M2 ion channel blockers are the most sensitive of all classes of influenza antiviral drugs; a single S31N change in the M2 channel protein provides a fitness-neutral, complete resistance, and is now near-universally present in influenza A infections, which is why the adamantanes have been permanently clinically obsolete (Deyde et al., 2007). In contrast to S31N, H275Y is not a universally transmitted resistance; with no fitness cost in the absence of permissive mutations, it was not transmitted until the D344N permissive mutation was established in the global 2007–2009 pandemic (nearly all transmission occurred on its own account), and then nearly disappeared when the genetically different virus A(H1N1)pdm09 became established (Brookes et al., 2011; Pizzorno et al., 2011). CENI resistance through the PA-I38 substitutions falls somewhere in between: the genetic barrier is also low (one substitution), fitness costs are measurable but less so than unmitigated H275Y, and confirmed community transmission has been reported, but limited to small local outbreaks, as is typical of a resistant lineage that has not yet evolved the necessary “background” mutations for unconstrained transmission (Takashita et al., 2019; Imai et al., 2020; Lee et al., 2021). The transmission potential of PB2 inhibitor resistance is completely uncharacterized because the drug was not taken forward into clinical trials, the resistance is found at nine different residues, more extensive than for the other three classes of which only one-residue bottlenecks are known. However, genetic barrier size alone is not a good predictor of transmission risk as it is fitness cost and subsequent availability of a permissive mutational pathway that determines transmission risk, therefore, alongside monitoring for primary resistance substitutions, surveillance should focus on monitoring for permissive mutations.

### 8.5 Combination therapy as a resistance mitigation strategy

Combination therapy, which uses drugs that target different, non-overlapping molecular processes, is a well-established technique for combating drug resistance. This technique has proven particularly effective in diseases such as HIV, hepatitis C, and tuberculosis, where combination regimens have reduced formerly difficult-to-treat infections to manageable conditions by raising the genetic barrier to resistance. In such instances, the virus or pathogen would have to evolve numerous resistance mutations at the same time, which is highly implausible within a single host. A similar rationale underpins influenza antiviral treatment. When drugs target different viral proteins, such as neuraminidase (NA) and cap-dependent endonuclease (PA), the virus would have to acquire resistance mutations in both targets simultaneously. Given that each mutation occurs with a low probability (about 10^-5^), the likelihood of both mutations happening in the same viral particle (around 10^-^¹^0^) is quite low. Because of this, it is extremely unlikely that dual resistance will develop inside a single host (Gubareva and Fry, 2020; Hussain et al., 2025).

Preclinical investigations have repeatedly shown that baloxavir-oseltamivir combination therapy is efficacious in a variety of experimental models. *In vitro*, the two drugs exhibit synergistic antiviral efficacy against both wild-type and PA-I38T baloxavir-resistant influenza A viruses in yield reduction experiments (Park et al., 2021). Notably, the highest synergy occurs at low baloxavir concentrations (0.006-1.56 nM), implying that clinically attainable drug doses are adequate to achieve this improved impact. *In vivo* data support this advantage. In mouse sequential lung-to-lung passage studies, treatment with baloxavir alone (1 or 5 mg/kg) resulted in the development of PA-I38X resistance mutations in 67% of mice. In contrast, combined therapy with baloxavir and oseltamivir at equivalent doses entirely prevented the formation of PA-I38X mutations across all passage generations (Koszalka et al., 2022a). These findings give direct experimental evidence that using dual-mechanism antiviral pressure can successfully reduce the emergence of resistance that often occurs with monotherapy. In addition, the preclinical mouse data demonstrate that baloxavir in combination with favipiravir or oseltamivir does not yield an increase in the lung viral load or in weight loss compared to either therapy alone, but does yield a survival improvement, suggesting that other endpoints that did not show an additive effect may show an additive survival benefit (Koszalka et al., 2022b).

It has been more difficult to translate this preclinical synergy into therapeutic benefit. Baloxavir marboxil administered to standard-of-care NAIs (oseltamivir, zanamivir, or peramivir) was compared to standard-of-care NAIs plus placebo in the FLAGSTONE phase 3 randomized controlled study, which included patients hospitalized with severe influenza (Kumar et al., 2022). Combination therapy did not improve clinical outcomes as determined by ordinal clinical status, time to clinical improvement, length of hospital stay, or mortality, despite significantly reducing the duration of culturable virus shedding compared to NAI monotherapy. This dissociation between virological and clinical benefit in hospitalized patients is similar to the pattern seen with other antivirals in severe disease and probably indicates that immunopathological host responses, rather than continuous viral replication, are primarily responsible for clinical deterioration at the time of hospitalization, a stage at which viral clearance has already started in the majority of immunocompetent patients (Gubareva and Fry, 2020; Kumar et al., 2022).

In addition to FLAGSTONE, there are two additional lines of clinical evidence that are informative. First, retrospective analyses of immunocompromised patients, especially patients receiving hematopoietic stem cell transplant (HSCT), have failed to show a clinical advantage for baloxavir-NAI combination therapy compared with monotherapy, and have consistently shown that combination therapy reduced the length of viral shedding, suggesting a virological-clinical dissociation, as seen in FLAGSTONE in the population with the highest theoretical need for combination therapy (Koszalka et al., 2022b). Second, a prospective randomized pilot trial specifically targeting allogeneic HSCT (AHSCT) recipients (NCT05170009) was disrupted by poor enrollment, suggesting a structural challenge to producing sufficient definitive evidence of combination therapy in the population where it would most be needed: AHSCT recipients have a higher risk for resistance, and also have a smaller number of patients than would be needed for adequate trial powering within standard trial timelines (Weill Medical College of Cornell University, 2025). This comes on top of the pimodivir-oseltamivir TOPAZ drug combination, which also failed to demonstrate efficacy during the Phase III trial (**Table 1**), and illustrates that achieving the benefit of combination synergy in the Phase III trial has not been possible in combination with other drug pairs, including baloxavir-NAI (Finberg et al., 2019; Koszalka et al., 2022b).

Although it reframes the clinical setting in which combination regimens are most likely to be beneficial, the negative FLAGSTONE clinical outcome result does not refute the molecular rationale for combination therapy as a resistance mitigation strategy. Combination therapy may be most effective in three particular clinical settings: immunocompromised patients with prolonged viral shedding and high treatment-emergent resistance rates who serve as the primary reservoir for resistant variant amplification and possible community dissemination; early outpatient treatment in high-risk patients before immunopathological amplification is established; and pandemic threat scenarios where an antiviral-naive population is exposed to a novel virus and maintaining drug efficacy through resistance prevention is of heightened public health importance (Gubareva and Fry, 2020; Kumar et al., 2022; Hussain et al., 2025). A logical extension of this approach is the creation of triple combination regimens that combine two direct-acting antivirals that target different viral proteins with a host-directed antiviral (like nitazoxanide or a MEK inhibitor). These regimens increase the genetic barrier to resistance while possibly offering additive antiviral and immunomodulatory benefits (Kumar et al., 2022).

### 8.6 Antiviral therapy in special populations: evidence gap

Pediatric, elderly, pregnant, immunocompromised, and critically ill patients are overrepresented in the burden of influenza-related morbidity and mortality, and are also underrepresented in the evidence base underlying current antiviral recommendations for dosing and treatment.

#### 8.6.1 Pediatric patients

The resistance picture in children is significantly different from that in adults. Up to 23.4% of children under 12 years of age have been found to be resistant to baloxavir, a much higher percentage than in adult cohorts, likely due to the increased selection pressure that occurs during treatment due to higher viral load and longer shedding periods during childhood. Resistance data is particularly limited in younger pediatric age groups, and a 2025 meta-analysis identified an increase in the proportion of pediatric isolates resistant to oseltamivir as well as growing use of baloxavir, with some countries continuing to recommend baloxavir for use only in children aged 5 years and older because of limited safety data in younger age groups (Hirotsu et al., 2023; Zhu et al., 2025).

#### 8.6.2 Pregnant and postpartum patients

Pregnant women are at higher risk of severe influenza and have been systematically underrepresented in antiviral RCTs, so there is no current guidance on dosage for pregnant women. There is limited information suggesting that oseltamivir and zanamivir are safe in pregnancy, with no safety or efficacy data available for peramivir, and baloxavir is contraindicated in pregnant or breastfeeding women because of the lack of safety and efficacy data in this population (Influenza in Pregnancy Prevention and Treatment; Chow et al., 2021).

#### 8.6.3 Elderly patients

In addition to the known immunosenescent effect on vaccine immunogenicity, older adults are underrepresented in antiviral clinical trials in general, which may be because of arbitrary upper age limits in clinical trial eligibility criteria. These raise questions about whether age-related pharmacokinetic or immunological shifts have a meaningful impact on antiviral activity or dosing in this group of individuals with the greatest absolute risk of influenza mortality (Cadar et al., 2023).

#### 8.6.4 Immunocompromised patients

It is consistently noted in the antiviral literature as the highest risk population for prolonged viral shedding and treatment-emergent resistant variants, and is a key reservoir for the amplification of resistant variants. However, there has been a relative lack of prospective antiviral trials in immunocompromised patients with the majority of evidence coming from case series, retrospective cohorts or from sub-analysis of trials in the general population.

#### 8.6.5 Critically ill patients

Pharmacokinetic information in the critically ill patient undergoing organ support is improving and is still limited. Standard dosage of oseltamivir seems to be sufficient in patients receiving ECMO alone, but in patients receiving both ECMO and continuous renal replacement therapy (CRRT), optimal dosage adjustment is not well established because oseltamivir carboxylate accumulates in patients with renal impairment. Currently, the dose of Peramivir in a setting of CRRT is based on individual case reports, but not on systematic pharmacokinetic studies (Eyler et al., 2012; Honore et al., 2020).

The common denominator is that, across all 5 populations, the dosing and treatment advice is based on trials in populations that this one is not close to. Future antiviral trial design will need to proactively ensure that special populations (such as pregnancy-specific or pediatric-specific pharmacokinetic studies) are included in order to close the gap that has been created until now, where they have been extrapolated in the *post-hoc* manner.

## 9 The H5N1 pandemic threat: stress-testing the current therapeutic arsenal

### 9.1 Clade 2.3.4.4b H5N1: virological characteristics relevant to treatment

A highly pathogenic avian influenza (HPAI) A(H5N1) has undergone a wide-ranging genetic evolution since its origin in the A/goose/Guangdong/1/1996 lineage, resulting in the formation of many clades and subclades due to constant genetic drift and reassortment (Webby and Uyeki, 2024; Xie et al., 2025). Among them, clade 2.3.4.4b has become the worldwide dominant one, spreading at the geographic scale never seen before since its resurgence in 2020 and its introduction to North America in late 2021 (Caliendo et al., 2022; Xie et al., 2025). As of January 2025, the World Health Organization has received 963 confirmed cases of human infection with H5N1 from 24 countries since 2003, which have resulted in 465 deaths and a cumulative case fatality rate (CFR) of about 48 percent, representing the highest case fatality rate of any influenza strain known to infect humans (Cumulative Number of Confirmed Human Cases for Avian Influenza a(H5N1) Reported to WHO, 2003-2025, 20 January 2025). Among the particularly clinical and virological features, it is marked by the extremely extended host range of the virus. In March 2024, the first case of clade 2.3.4.4b was reported in dairy cattle in the United States, which had not previously been reported in a bovine host (FAO/WHO/WOAH, 2025). Epidemiological studies have identified two major genotypes that cause persistence in epidemics: B3.13, which is a dominant strain in dairy cattle and is a complex reassortant between Eurasian HPAI H5N1 strains and low-pathogenicity avian influenza viruses of North American origin (D1.1) (Nguyen et al., 2025). As of July 2025, more than 1,074 dairy cattle herds in 17 states in the US tested positive for H5N1, with spillover events documented in over 43 mammalian species across Europe, North America, South America, and Asia (Chrzastek et al., 2025; FAO/WHO/WOAH, 2025). Treatment-wise, one of the key structural aspects of the existing 2.3.4.4b virus’s clade is the affinity to the receptor. Recent surveillance findings indicate that circulating strains still have preferential affinity to avian-like a2,3-linked sialic acid receptors, as opposed to the a2,6-linked receptors found in the human upper respiratory tract (Webby and Uyeki, 2024; Chrzastek et al., 2025). This explains why most of the contemporary human infections that have been spread in direct contact with animals as opposed to human to human. Nonetheless, this attribute can be altered; experimental evidence has shown that a single point mutation(Q226L) in the bovine-origin H5N1 hemagglutinin may alter receptor specificity to human-type receptors, and the implications of this finding are immense on the risks of pandemics as well as the tissue tropism that determines responses to treatments (Lin et al., 2024). This issue was demonstrated clinically by the case of a critically ill Canadian teenager in late 2024, where lower-airway isolates sequencing revealed mutations E627K of the polymerase basic 2 (PB2) gene, E186D and Q222H of the H5 hemagglutinin gene, which are associated with increased virulence and partial adaptation to mammals (Jassem et al., 2025). These virological characteristics also determine the clinical manifestation of severe cases of H5N1. The rapid progression of pneumonia and acute respiratory distress syndrome (ARDS) seen in response to the high replicative efficiency in the lower respiratory system are features that directly limit the time window available to effective treatments with antiviral agents. The disease manifests (severe infection) that includes pneumonia, multiorgan failure, lymphopenia, and high levels of aminotransferases, which explains why the treatment should be started as soon as possible (Garg et al., 2025). The continued reassortment potential of clade 2.3.4.4b, evidenced by the contemporarily exhibiting genetically distinct genotypes of a number of host-specific species, further complicates the idea of future antiviral susceptibility profiles, necessitating annual phenotypic monitoring in order to guide treatment policy (Pascua et al., 2025).

### 9.2 Antiviral susceptibility of H5N1 to all drug classes: current surveillance data

The surveillance data as of 2023–2025 show a fairly encouraging picture of retained vulnerability of the key approved antiviral drug classes, although there are critical details that should be carefully considered (**Table 3**).

**Table 3:** H5N1 Clade 2.3.4.4b Antiviral Therapeutic Landscape 2025.

Agent	Highest evidence level for H5N1	H5N1 Clade 2.3.4.4b susceptibility	Human RCT data for H5N1	Animal model efficacy	Current CDC/WHO recommendation	Global stockpile status	Critical evidence gaps required for practice change
Approved antivirals							
Oseltamivir (Tamiflu)	Level III	Susceptible (IC_50_: subnanomolar– low nanomolar). ~4× less potent vs. clade 2.3.4.4b than vs. 2.3.2.1c	NONE (observational registry only; n=113 in multi-country H5N1 registry)	Positive (ferret, mouse models; early treatment critically important)	First-line	Primary stockpile agent globally; SNS (US); WHO global stockpile	RCT data in H5N1 infection. Optimal dose and duration in critically ill patients. Pharmacokinetics on ECMO/CRRT. Whether higher doses (150 mg BID) confer added benefit in H5N1 vs seasonal dose
Peramivir (Rapivab)	Level III	Susceptible (IC_50_: subnanomolar; no significant geographic variation in 2024 data)	NONE (no H5N1-specific RCT; inferred from seasonal IV data)	Limited H5N1-specific animal data	First-line (IV)	Limited stockpile; not in WHO global stockpile	Formal H5N1-specific clinical study. Pharmacokinetics in H5N1-associated multi-organ failure. Availability in LMIC settings
Baloxavir Marboxil (Xofluza)	Level IV	Susceptible (EC_50_: low nanomolar; no PA-I38X detected in 2023–2024 H5N1 human isolates)	NONE (zero published human H5N1 treatment outcomes with baloxavir as primary therapy)	POSITIVE (Andreev et al., 2026; Belser et al., 2026)	Expert opinion (limited data)	NOT included in major national stockpiles for mass treatment; small quantities only	Human H5N1 RCT or adaptive trial. Optimal dosing and duration in H5N1 (higher dose needed)?. Whether combination with oseltamivir improves H5N1 outcomes over monotherapy. PA-I38X emergence rate in H5N1 patients treated with baloxavir
Zanamivir IV (investigational)	Level III	Susceptible (retains activity vs. H275Y; strongest of all NAIs vs. resistant mutants)	Phase 3 in seasonal influenza (Marty et al., 2017); NO H5N1-specific data	Positive in seasonal/H5N1 models	Consider (expert opinion only)	Not stockpiled in most countries; production limited	H5N1 clinical trial data. Manufacturing scale-up for pandemic scenarios. Comparative efficacy against peramivir in H5N1
Amantadine/Rimantadine	Level IV	Susceptible *in vitro* (unlike seasonal H3N2; 2 Cambodian clade 2.3.2.1c isolates resistant in 2024)	NONE	Historical positive in early H5N1 animal studies; no modern clade 2.3.4.4b data	Not recommended (clinical use)	Available in SNS; not endorsed for H5N1 treatment use	No priority for H5N1 clinical investigation given rapid resistance emergence risk. Documented only as emergency adjunct (BC adolescent case 2024)
Investigational & host-directed agents							
Pimodivir (VX-787; PB2 inhibitor)	Level IV	Susceptible (EC_50_: low nanomolar in 2023–2024 H5N1 panel; no PB2 CBD resistance markers detected in 2.3.4.4b)	NONE (Phase 3 development discontinued for seasonal influenza; no H5N1 trials)	Positive vs. H5N1, H7N9, H5N6, H4N2 (Patel et al., 2021)	Investigational	Not stockpiled	Phase 2/3 clinical data in H5N1. Combination pimodivir + oseltamivir or baloxavir in H5N1 animal models with clade 2.3.4.4b. Regulatory pathway for emergency use authorization
Corticosteroids (immunomodulatory)	Level II (harm signal)	N/A (not antiviral)	No RCT for H5N1; observational data from H1N1/H5N1: associated with increased mortality and nosocomial infection	N/A	Not recommended (clinical use)	N/A	H5N1-specific RCT. Role in ARDS management per individual clinical course. Biomarker-stratified trials to identify subgroup (e.g., HLH/MAS) that may benefit
Combination Therapy (Oseltamivir + Baloxavir)	Level IV (preclinical only for H5N1)	Both agents susceptible (see above)	NONE (FLAGSTONE trial: negative in seasonal severe influenza)	POSITIVE (Belser et al., 2026)	Expert opinion (limited data)	Operationally impossible at scale until baloxavir stockpiled	H5N1-specific combination trial. Whether virological benefit in H5N1 translates to clinical outcome benefit (unlike in seasonal hospitalized influenza). Optimal dosing, timing, and duration for H5N1 combination therapy

#### 9.2.1 Neuraminidase inhibitors: current evidence

In neuraminidase inhibition (NI) experiments on H5N1 viruses recovered in human cases during 2023-2024, all strains in clade 2.3.2.1c (Cambodia) and clade 2.3.4.4b (Americas) were sensitive to oseltamivir, zanamivir, peramivir, laninamivir and the investigational drug AV5080 with an IC50 in the range of subnanomolar to low nanomolar (Pascua et al., 2025). An analysis with clinical implications, though, is that the neuraminidase activity of clade 2.3.2.1c viruses was approximately 4-fold more potently inhibited by the neuraminidase inhibitor, oseltamivir, than that of clade 2.3.4.4b viruses, a facet of the protein which is attributed to amino acid variation in the neuraminidase protein (Pascua et al., 2025). This difference in potency is not resistance as per phenotypic criteria, but it creates a concern regarding the optimal dose and increases the hypothetical limit of the development of frank resistance in clade 2.3.4.4b infected people.

H5N1 isolates of dairy cattle in the US, including those of Texas and Ohio, also tested phenotypically, and were all susceptible to all three NAIs at concentrations below nanomolar, with no large geographic differences (Andreev et al., 2024a). Analysis of 202 NA gene sequences of the 2024 bovine outbreak in the US found only a single sequence with the NA-T438I substitution (N1 numbering), which had been previously reported to cause decreased zanamivir and peramivir inhibition (Andreev et al., 2024a). In 2022-2023, a larger genotypic survey on the global distribution of circulating clade 2.3.2.1a and 2.3.4.4b viruses showed that substitutions with reduced NAI inhibition occurred only in 0.78% of 2,698 examined sequences, which is a strong reassurance that resistance has not entered the mainstream (Andreev et al., 2024b).

However, there have been occasional reports of H5N1 viruses resistant to oseltamivir- and baloxavir-resistant monitoring systems in wild birds, including in the 2.3.4.4b clade and drug-resistant variants are a known risk that has emerged especially when young children and immunocompromised patients are being treated (Pascua et al., 2025). Further, certain viral isolates of clade 2.3.4.4b have acquired mutations that change the drug-binding sites and cause resistance by structural alteration instead of the traditional H275Y substitution route (Nguyen et al., 2023). These results strongly point to the need to conduct real-time phenotypic surveillance with genomic monitoring.

#### 9.2.2 Cap-dependent endonuclease inhibitors: baloxavir and next-generation agents

All H5N1 viruses tested in 2023-2024, both of clade 2.3.2.1c and 2.3.4.4b, are susceptible to baloxavir marboxil and the experimental drug tivoxavir with 50 percent effective concentrations (EC50) in the low nanomolar range (Pascua et al., 2025). The molecular markers of an observed association with a low susceptibility to baloxavir were not found in polymerase acidic (PA) genes sequences of human-derived 2023–2024 H5N1 viruses (Pascua et al., 2025). Rates of PA replacements linked with the lower frequency of baloxavir inhibition in the 2022–2023 global surveillance database constituted 0.54% (14 of 2,600 sequences), which is very similar to the incredibly low rate of NAI resistance markers (Andreev et al., 2024b).

#### 9.2.3 Adamantanes (M2 ion channel blockers): retained *in vitro* susceptibility

Unlike seasonal influenza A (H3N2), which has near-universal adamantane resistance, current clade 2.3.4.4b H5N1 viruses retain *in vitro* susceptibility to M2 ion channel blockers amantadine and rimantadine, with the exception of two clade 2.3.2.1c isolates from Cambodia that showed resistance in recent phenotypic tests (Pascua et al., 2025). Despite this residual susceptibility, amantadine and rimantadine have no therapeutic value in the treatment of H5N1 illness. This is due not to inherent resistance, but to the well-known risk of rapid *de novo* resistance emergence under selective drug pressure, the lack of clinical benefit data, and the exclusion of this drug class from all current guidance on the treatment of novel influenza A virus infections in humans (Pascua et al., 2025; CDC, 2026b). The British Columbia adolescent in 2024 was the only documented case in which amantadine was added to a treatment plan. This was an emergency combination decision motivated by the severity of the critical illness, started prior to the availability of formal susceptibility data, and should not be taken as proof of therapeutic benefit or as a model for routine use (Jassem et al., 2025).

#### 9.2.4 Investigational and pipeline agents

Other than licensed drugs, the polymerase basic 2 (PB2) polymerase inhibitor pimodivir was found susceptible to all H5N1 viruses tested with EC50s in low nanomolar ranges in the study series of 2023–2024 as was the experimental compound tivoxavir (in Phase 1 trials) (Pascua et al., 2025). These data widen the theoretical arsenal but are yet to be validated in clinical studies of H5N1 infection.

### 9.3 What therapeutic options exist today for severe H5N1 infection?

The treatment regimen of severe H5N1 infection in 2025 will be based on the humble model of one oral antiviral approved and whose use in humans has been observed to reduce mortality, one intravenous H5N1 alternative to those who cannot tolerate the oral form, one more approved drug category, which has promising *in vitro* data but no clinical data, and a set of supportive care interventions stolen out of general critical care. No randomized controlled trial data support the use of immunomodulatory therapy for H5N1.

#### 9.3.1 Oseltamivir

Oseltamivir is still the pillar in the recommended treatment of H5N1 infection. Recent CDC guidelines advise a standard dose of 75 mg twice each day of oseltamivir 5 days of use in adolescents and adults, with the recommended dose initiated as soon as possible of symptom onset, in both outpatients and hospitalized patients with suspected, probable and confirmed infection (CDC, 2026b). Evidence supporting such a recommendation is based mainly on observational data but not randomized trials. In a multi-country observational registry of HPAI A (H5N1) patients, the initiation of treatment with oseltamivir within 2 days of the onset of symptoms, in patients who had not yet developed respiratory failure at the time of treatment initiation, was significantly related to survival (n = 113) (Adisasmito et al., 2010; CDC, 2026b). This observation is biologically likely: early treatment decreases viral replication at the stage at which lower respiratory tract viral load causes the immunopathological cascade resulting in ARDS. On the other hand, late treatment, started when respiratory failure is already present, has not shown a uniform survival advantage, and this parameter plays the primary role of immunopathology compared to the persistence of viral replication during the late phase of the disease (Pascua et al., 2025). In the case of critically ill patients who are unable to tolerate or absorb enteral oseltamivir because of gastric stasis, malabsorption, or gastrointestinal bleeding, intravenous peramivir (600 mg once daily at least 5 days in hospitalized patients) should be used instead (CDC, 2026b). Absorptive problems are also of clinical significance in H5N1 as critically ill patients often need intensive care treatment with a diminished gastrointestinal motility. According to available data, the orally administered or naso/orogastrically administered oseltamivir maintains adequate oral bioavailability even in patients with continuous renal replacement therapy, or extracorporeal membrane oxygenation (ECMO) as the clinical judgment is required due to individual pharmacokinetic variability of the medication (CDC, 2026b).

#### 9.3.2 Baloxavir marboxil

Baloxavir is licensed to be used in the treatment of uncomplicated seasonal influenza but was not tested in any clinical research in patients with H5N1 or other new influenza A virus infections (CDC, 2026b). In mice and ferrets, animal trials indicate clinical efficacy of early baloxavir treatment in animal viral infections of HPAIV but the dosing, duration, and clinical extrapolation of these results are not well understood (Andreev et al., 2026; Belser et al., 2026). Combination therapy with baloxavir and oseltamivir was used in the clinical case of the critically ill British Columbia adolescent, who was among the few reported cases of severe clade 2.3.4.4b infection in a patient in North America, after reaching a critical illness, serial PCR testing demonstrated to decrease the viral loads after combination therapy but the effect of each drug cannot be separated in this isolated case (Jassem et al., 2025). Expert opinion on baloxavir (especially in combination with oseltamivir) should be taken into account in hospitalized or immunocompromised patients with H5N1 infection since the susceptibility *in vitro* and data on animal models indicate that baloxavir may be helpful, although no clinical trial results have been published (Pascua et al., 2025; Belser et al., 2026).

#### 9.3.3 Combination antiviral therapy

Animal studies have shown that combination therapies that attack various phases of the influenza replication cycle are beneficial compared to single-agent therapy against H5N1. Combinations of agents like oseltamivir and baloxavir have been shown to be more effective in animal models in relation to viral replication and survival rates as opposed to each agent alone (Belser et al., 2026). The argument is more than mere efficacy: by attacking two independent mechanisms at once the selection pressure to evolve any form of resistance is decreased, an argument of special relevance in the view of the known tendency of the influenza RNA polymerase to produce variants during replication. Combination therapy has to be considered in patients that are immunocompromised or hospitalized, pending clinical data on optimal dosing, duration, and regimen selection for H5N1-specific settings must be obtained (Belser et al., 2026).

#### 9.3.4 Critical care and supportive management

In addition to antiviral agents, organ support remains a critical determinant of severe H5N1 infection management. This necessitates ventilatory support of severely ill H5N1 patients who are in the hospital within 48 hours of hospitalization and in most cases in ARDS with multiorgan failure (Abdel-Ghafar et al., 2008). This teenager in British Columbia needed tracheal intubation followed by ECMO instituted on a venovenous and continuous renal replacement therapy (CRRT) (Jassem et al., 2025). There is experience of ECMO in H5N1 patients as a bridge to recovery, similar to its experience in severe ARDS of other etiologies, but no specific outcome data of a controlled population of H5N1 patients undergoing ECMO. The use of corticosteroids in severe H5N1 infection remains unresolved. Observational data and systematic review evidence from influenza-associated ARDS indicate that corticosteroid therapy is associated with increased mortality and higher rates of nosocomial infection, with mechanistic concern that immunosuppression may prolong viremia (Ni et al., 2019). In the absence of H5N1-specific trial data, corticosteroid decisions must be guided by the individual clinical course and the broader critical care literature on ARDS management (CDC, 2026b).

### 9.4 Gaps in the evidence base: why RCT data for H5N1 antivirals is absent

The lack of randomized controlled trial (RCT) data on antiviral treatment of H5N1 infection does not indicate any lack of investigation, but is an epidemiological and logistical fact that is unlikely to be addressed before the next pandemic itself provides the population of patients that would be needed to undertake such trials.

The basic limitation is the scarcity of human cases. Of the approximately 25 years between 1997, with the first report of H5N1 as a zoonotic threat, and 2024, there has been less than 1,000 known cases of human infection around the globe annually of this pathogen on average (Cumulative Number of Confirmed Human Cases for Avian Influenza a(H5N1) Reported to WHO, 2003-2025, 20 January 2025). These instances are infrequent, in dozens of countries, in situations where the treatment window is narrowed due to a rapid clinical deterioration. Since the published literature has been explicitly noted to show that it has not conducted sufficient enrolled RCTs to determine the effectiveness of antiviral therapy or postexposure prophylaxis in any patients having new infections with influenza A virus related to severe human disease (Uyeki and Belser, 2025). Having found this conclusion after the whole-hearted search of evidence up to March 2025, it is important to state that it is impossible to conduct the conventional RCTs in the present conditions of epidemiology.

The second limitation is the ethical aspect. Since there is observational evidence of early initiation of oseltamivir correlating with survival in patients with H5N1 (CDC, 2026b), and the disease has a very high initial baseline CFR, withholding what appears to be a viable treatment, in one arm of the trial, creates serious ethical challenges to trial design. This difficulty is likened to the one faced in other quickly lethal emerging infections because equipoise has not been easy to implement in practice.

Third, H5N1 genotype emergence remains unpredictable with B3.13 and D1.1 strains currently circulating with dissimilar phenotypic characteristics, which implies that clinical evidence developed on a single genotype could not be easily transferred to others (FAO/WHO/WOAH, 2025; Nguyen et al., 2025). This 4-fold difference in the potency of the oseltamivir virus against clade 2.3.4.4b and 2.3.2.1c viruses (Pascua et al., 2025) is an indication that the data of antiviral treatment may not be widely applicable even within the genus H5N1. Any future strain of the pandemic may have further deviated in reference to the strains against which *in vitro* and animal model data are presently available.

Fourth, available data of seasonal and pandemic influenza of 2009 only has partial information on severe H5N1 disease. The usual standard dosing, neuraminidase inhibitor RCTs of seasonal influenza used outcomes of time to symptom relief, which are not easy to extrapolate to a disease with a 48% past CFR and early and rapid progression to respiratory failure. Limited clinical pharmacokinetic data of critically ill patients, including those on ECMO or CRRT, is available and renal and hepatic impairments in severe cases of H5N1 do not predict the disposition of drugs in a manner that has been reflected in licensing data (Wong et al., 2025; CDC, 2026b). Clinical trials of double dose oseltamivir in hospitalized seasonal influenza showed no significant effect, but the same effect has not been investigated on H5N1, with its different pathophysiology (CDC, 2026b).

The lack of human clinical data of baloxavir in H5N1 is particularly an example of such a gap. Although *in vitro* data on susceptibility and positive results in preclinical animal models, no published treatment outcome data of any human case of H5N1 in which baloxavir was utilized as the primary therapy is shown (CDC, 2026b). The new mechanism of action, convenient single-dose, and quick clearance of viruses in seasonal influenza are logically appealing features of the drug, but cannot replace empirical human data and its optimal function, whether as monotherapy or in combination with other drugs, dose and duration of action, has not been characterized at all in this clinical scenario.

This series of gaps, including no RCT, scanty case series, observational data that is largely based on previous clades, no pharmacokinetics in critically ill patients and no human data on the new agents, is what defines the evidence gap within which the clinical decision-making of severe H5N1 currently functions. The most promising mechanisms, in which this evidence base could be created in real time during a future pandemic event, are adaptive clinical trial frameworks, pre-negotiated master protocols, and platform trial infrastructure.

### 9.5 Stockpiling strategy and therapeutic preparedness for H5N1 pandemic scenarios

With these therapeutic uncertainties discussed above, national and global pandemic preparedness models have highlighted antiviral stockpiling as the main near term countermeasure while vaccine and novel therapeutical development is underway. The explanation is simple: antivirals are the only registered, quickly implemented pharmaceutical interventions that exist during the crucial first months of an outbreak pandemic, when it is not possible to produce vaccines in large quantities.

#### 9.5.1 United States: Strategic National Stockpile and BARDA

Millions of treatment courses of antiviral drugs are stored in the US Strategic National Stockpile (SNS), and the backbone of antiviral component is the oseltamivir (Tamiflu) (ASPR’s Response to H5N1 Bird Flu). The Administration for Strategic Preparedness and Response (ASPR) has committed to distributing SNS oseltamivir to jurisdictions that do not have their own stocks when a declared public health emergency occurs (ASPR’s Response to H5N1 Bird Flu). The Biomedical Advanced Research and Development Authority (BARDA) is in charge of the National Pre-Pandemic Influenza Vaccine Stockpile (NPIVS), which was created in 2005 and aimed to hold stockpiled vaccines that would inoculate 20 million critical-workforce personnel at the initial stages of a pandemic response (Objective 1: Strategic Implementation and Deployment of the U.s. National Pre-Pandemic Influenza Vaccine Stockpile (NPIVS)). CDC has developed several H5N1 candidate vaccine viruses (CVVs) that are almost identical to the hemagglutinin protein of recently circulating clade 2.3.4.4b viruses and have been distributed to vaccine manufacturers in the event of possible deployment (CDC, 2026c).

In May 2024, BARDA signed with CSL Seqirus to fill around 4.8 million more doses of bulk H5N1 vaccine antigen in vials to expedite physical availability of the stockpile to fill-finish operations (Objective 1: Strategic Implementation and Deployment of the U.s. National Pre-Pandemic Influenza Vaccine Stockpile (NPIVS)). As of 2025, concurrent clinical trials to assess candidate H5N1 vaccines adjuvated with MF59 and AS03 are in progress to produce the regulatory licensure evidence necessary to roll out stockpiled vaccines with regulatory confidence (Objective 1: Strategic Implementation and Deployment of the U.s. National Pre-Pandemic Influenza Vaccine Stockpile (NPIVS)). BARDA is also seeking to include mRNA-based pandemic influenza vaccines in the NPIVS, and the US Department of Health and Human Services had committed about $590 million to Moderna to speed up the creation of mRNA-based pandemic influenza vaccines (ASPR’s Response to H5N1 Bird Flu).

#### 9.5.3 Global level: WHO antiviral stockpile and the i-MCM-net

The outbreak of the SARS and the H5N1 infections of 2003–2004 led to the creation of a global influenza antiviral stockpile by the WHO, which was effectively operationalized during the 2009 A(H1N1) pandemic and allowed the provision of antiviral drugs to 72 countries within a short period of time (Shindo et al., 2025). The 2011 Pandemic Influenza Preparedness (PIP) Framework streamlined plans of equal access and supply chain preparedness. The experiences of the COVID-19 pandemic with the uneven distribution of vaccines resulted in the formation of the Interim Medical Countermeasures Network (i-MCM-net) led by WHO that facilitates integrated supply chains, real-time information exchange, and coordinated access to medical countermeasures such as antivirals (Shindo et al., 2025) Availability of Oseltamivir in low- and middle-income countries has also been expanded through the WHO Model List of Essential Medicines and the Prequalification Programme by acknowledging that equitable access to antivirals is one of the most important predictors of pandemic mortality globally (Shindo et al., 2025).

WHO clinical guidelines still recommend the use of the first-line treatment of the H5N1 infection with oseltamivir and stress the necessity of having appropriate antiviral stocks to treat the infection promptly (World Health Organization, 2024; Shindo et al., 2025). In the 2025 WHO Pandemic Agreement (World Health Assembly Adopts Historic Pandemic Agreement to Make the World More Equitable and Safer From Future Pandemics) adopted by the 78th World Health Assembly, new principles of equitable access to pandemic countermeasures such as antivirals are presented, and it is set to address the existing access inequities that occurred during the COVID-19 pandemic.

#### 9.5.3 Key lapses in stockpiling strategy

Despite such preparedness investments, there are multiple vulnerabilities in the structure. To begin with, the stockpiling plan has been overly focused on oseltamivir, which has a more restricted evidence basis of observational data and which appears to be less potent against clade 2.3.4.4b as compared to other clades (Pascua et al., 2025). Baloxavir, having a complementary mechanism of action and possibly better viral clearance kinetics, are not included in the vast majority of national antiviral stockpiles such as to be able to allow mass treatment in a pandemic. Second, the logistics involved in stockpiled antivirals have challenges such as shelf life, which necessitates the need to have replacement cycles and quality monitoring programs so that the stockpiled drugs are said to be good at the time they are used (Jones et al., 2023). Third, the stockpiling model is not yet sufficiently looking at the risk of resistance development in use of the stocks in case of a pandemic just as it was the case with the oseltamivir-resistant H1N1 strain in 2007-2009; a pandemic precipitated by a partially resistant H5N1 strain would quickly diminish the value of stockpiles that consist of a single drug class (Dixit et al., 2013; Jones et al., 2023). Fourth, equity in the antiviral stocks in the world has not been established: WHO structures offer a framework through which the low- and middle-income countries may access, but the capacity and cold-chain facilities to deploy these medications in resource-strained conditions within a pandemic outbreak have not been properly evaluated (Shindo et al., 2025).

**Figure 7:**
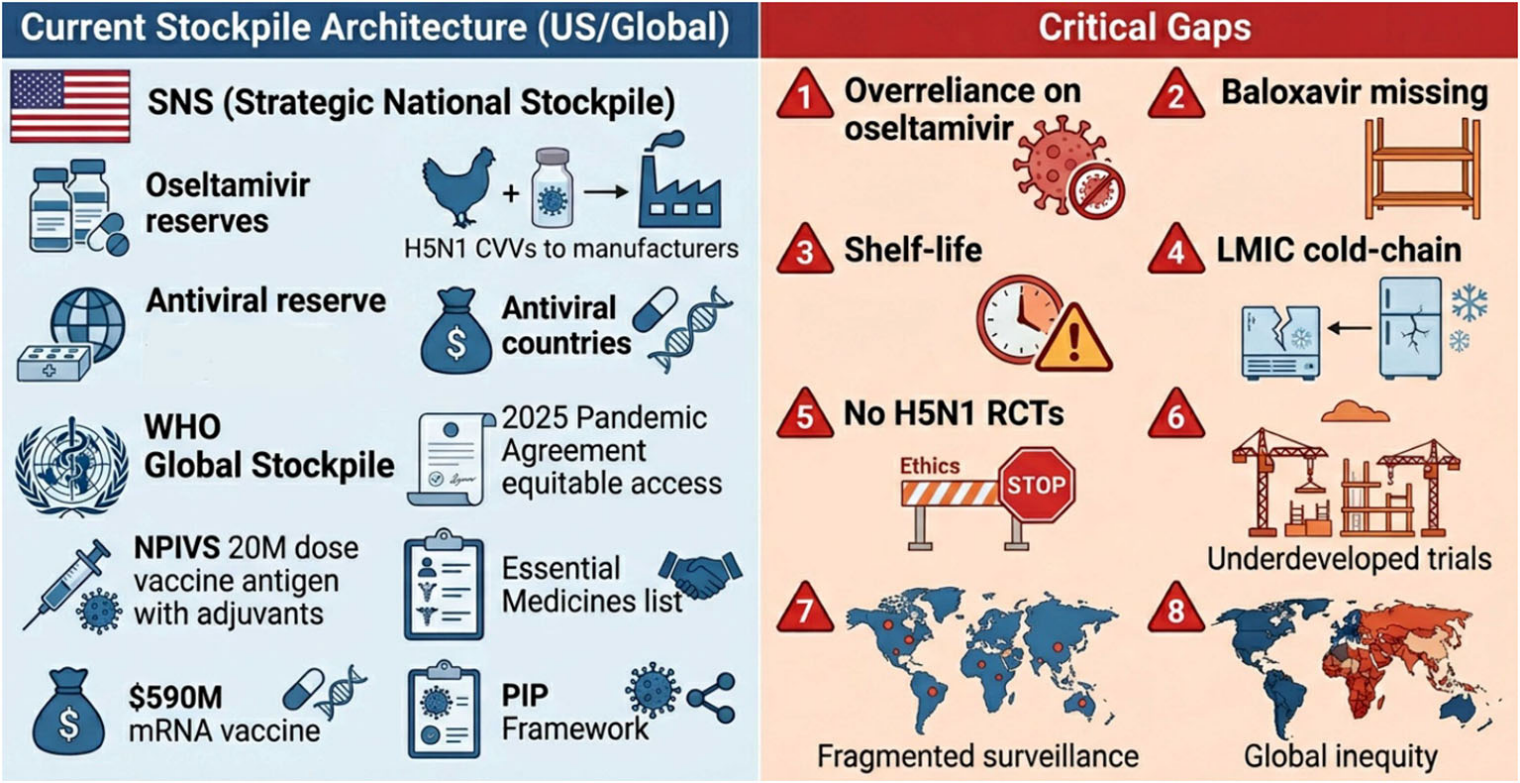
Current antiviral stockpile architecture and critical preparedness gaps for H5N1 pandemic response. Left: key components of US and global stockpile infrastructure, including oseltamivir reserves, H5N1 candidate vaccine viruses, the National Pre-Pandemic Influenza Vaccine Stockpile, mRNA vaccine investment, the WHO global stockpile, the 2025 Pandemic Agreement, the Pandemic Influenza Preparedness Framework, and the WHO Essential Medicines List. Right: eight critical gaps in current preparedness, including overreliance on oseltamivir, the absence of baloxavir from major national stockpiles, shelf-life and cold-chain challenges in low- and middle-income countries, the absence of H5N1-specific randomized trial data, underdeveloped adaptive trial infrastructure, fragmented surveillance, and persistent global inequity in antiviral access.

In 2025, the Global Virus Network (GVN), a group of virologists in more than 40 nations published an in-depth analysis, according to which governments should set plans to roll out therapeutics in case of an emergency to provide antivirals, which is a key part of pandemic preparedness, alongside structures to conduct real-time clinical studies on novel strain of the virus and therapeutic interventions (Bartlett et al., 2025). The disjointed state of modern surveillance, information-sharing, readiness planning, illustrates a discrepancy between the hypothetical accessibility of countermeasures and the practical implementation of them on a pandemic scale (Bartlett et al., 2025) (**Figure 7****).**

Overall, therapeutic preparedness in 2025 in response to H5N1 pandemic can be represented by the stockpile which is based on one single partially-evidenced drug group, the promising new agent (baloxavir) of which H5N1-specific clinical data are completely lacking, the emerging combination therapy options which have been validated in preclinical models alone, and the critical care support which is the primary line of response to the severe disease. A combination of expansion of stockpiles and synchronized international investment in adaptive trial facilities, real-time pharmacovigilance, and equal distribution systems are needed to bridge these gaps and work at a pace in the face of pandemic.

## 10 Emerging antiviral platforms and future directions

### 10.1 CRISPR-Cas13 and RNA-targeting antivirals

**Figure 8:**
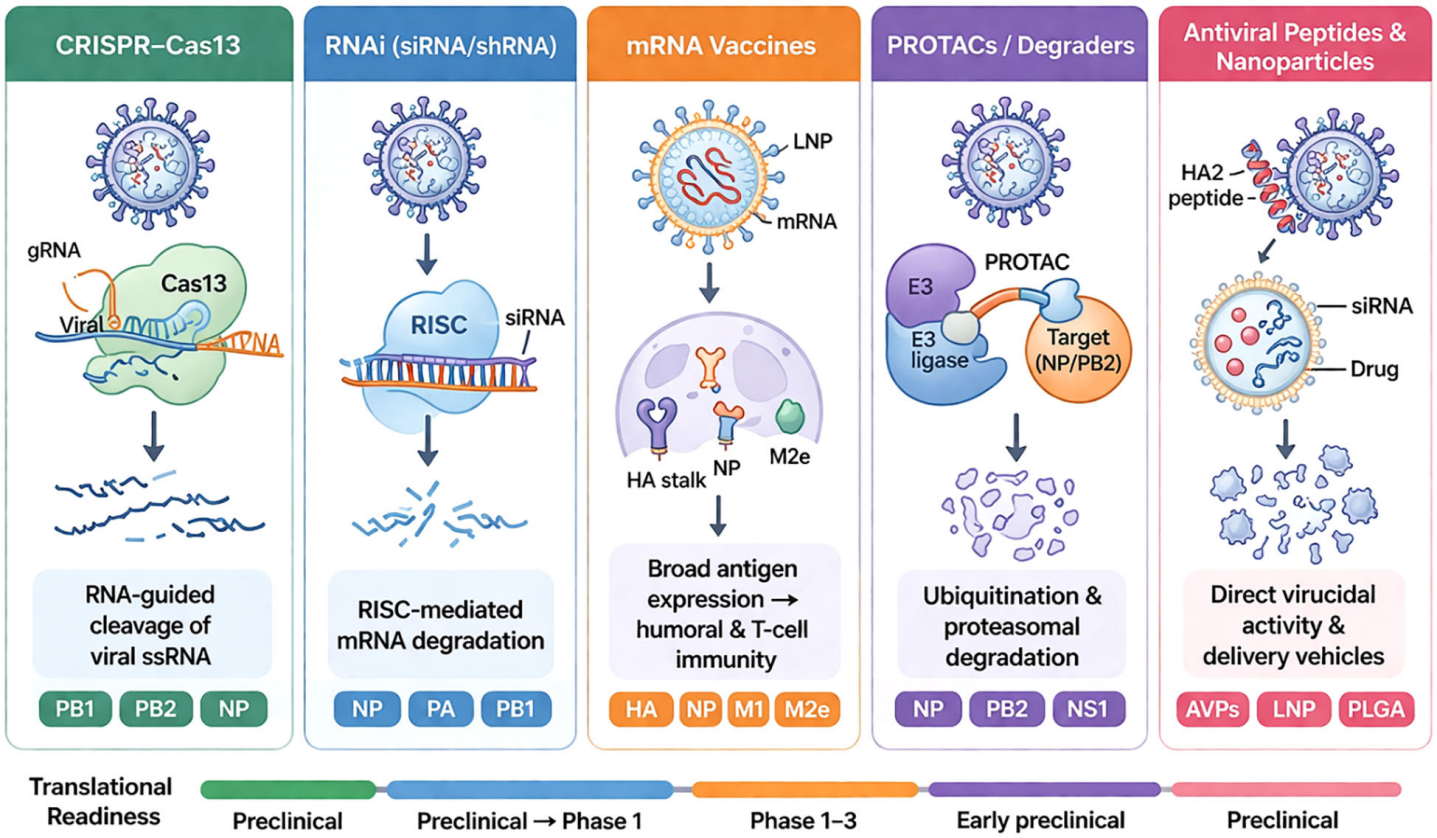
Mechanisms of action and translational readiness of emerging antiviral platforms against influenza virus. Five platform categories are depicted: CRISPR-Cas13 RNA-guided cleavage of viral ssRNA; RNAi-mediated degradation of viral mRNA; mRNA vaccine platforms encoding conserved antigens for broad humoral and T cell immunity; PROTAC-mediated degradation of viral proteins; and antiviral peptides and nanoparticle delivery vehicles. A translational readiness bar indicates the current clinical development stage of each platform.

The type VI effector Cas13 only recognizes and cleaves single-stranded RNA, which makes it perfect for directly interfering with RNA virus genomes without changing the host genome in any other way. Cas13 could be used to program RNA editing, which laid the biochemical groundwork for its use as an antiviral (Cox et al., 2017) and the compact Cas13d orthologue (CasRx), which exhibited superior knockdown efficiency among Cas13 family members and an improved safety profile due to its minimal collateral cleavage activity (Konermann et al., 2018)(**Figure 8**).

The CARVER (Cas13-Assisted Restriction of Viral Expression and Readout) platform achieved potent inhibition of lymphocytic choriomeningitis virus, influenza A virus, and vesicular stomatitis virus in human cell culture, underscoring its broad-spectrum capability (Freije et al., 2019). The related PAC-MAN (Prophylactic Antiviral CRISPR in huMAN cells) showed that Cas13d guide RNAs targeting conserved regions of the influenza A polymerase complex could suppress H1N1 replication in human lung epithelial cells and that a small number of guide RNAs could target all coronaviruses, which shows that the method can be used on a larger scale to prepare for a pandemic (Abbott et al., 2020).

The key remaining challenge is *in vivo* delivery. Nebulized, mRNA-encoded Cas13a in polymer-based nanoparticles reduced influenza A viral burden in mouse lung tissue and decreased SARS-CoV-2 replication in rodents, with the transient, non-integrating nature of mRNA delivery offering a meaningful safety advantage (Blanchard et al., 2021). The immunogenicity of bacterial-derived Cas13 proteins, the stability of guide RNA in the respiratory mucosa, and the potential presence of pre-existing anti-Cas antibodies in human populations and dose-dependent collateral cleavage all remain active areas of investigation; upgrades in guide RNA chemistry, such as 2′-O-methyl and phosphorothioate backbone changes, are expected to improve stability and reduce innate immune detection ahead of first-in-human studies.

### 10.2 RNA interference (siRNA and shRNA) therapeutics: delivery challenges

RNA interference (RNAi) was identified as a strong sequence-specific gene-silencing mechanism in mammalian cells, mediated by synthetic 21-nucleotide RNA duplexes that catalytically degrade complementary mRNA transcripts (Elbashir et al., 2001). Its antiviral potential was demonstrated soon after: systemic delivery of siRNAs targeting conserved influenza A segments, including the nucleoprotein (NP) and acidic polymerase (PA) subunit, provided significant protection against lethal viral challenge in murine models (Ge et al., 2004; Tompkins et al., 2004). These important investigations proved that RNA-mediated gene silencing could be used as an effective antiviral strategy *in vivo* (**Figure 8**).

Clinical translation has been limited by siRNA’s intrinsic delivery problems: its enormous molecular weight, polyanionic charge, susceptibility to nuclease breakdown in serum, and rapid renal clearance preclude passive cellular uptake and necessitate specialized carriers (Setten et al., 2019). Unmodified siRNA is neutralized in bodily fluids within minutes, and endosomal trapping after uptake remains a further bottleneck limiting cytoplasmic transport (Bobbin and Rossi, 2016). Chemical modifications including 2′-fluoro, 2′-O-methyl, and locked nucleic acid replacements, substantially increase nuclease resistance and reduce off-target immunostimulation via toll-like receptors 3, 7, and 8. Patisiran, a lipid nanoparticle-formulated siRNA targeting transthyretin mRNA demonstrated the clinical viability of systemic siRNA therapy in humans, but its liver-targeted delivery exploits hepatotropic nanoparticle uptake that does not translate to the pulmonary epithelium, the primary site of influenza replication (Adams et al., 2018).

Kulkarni et al. described the present state of nucleic acid treatments and identified ionizable lipid nanoparticle (LNP) chemistry as the most clinically advanced delivery platform for RNA payloads (Kulkarni et al., 2021). For respiratory antiviral uses, inhaled siRNA formulations are an appealing delivery route because they avoid systemic exposure and concentrate the treatment at the site of infection. Setten and colleagues documented a variety of developing techniques, including GalNAc conjugation for hepatocyte targeting, dynamic polyconjugates, and exosome-based carriers, to demonstrate the breadth of the delivery innovation landscape (Setten et al., 2019). The benefit of sustained gene silencing is provided by short hairpin RNA (shRNA), which is expressed intracellularly from viral or non-viral DNA vectors and converted into siRNA by the endogenous Dicer enzyme. However, rigorous safety profiling is required due to concerns about vector immunogenicity, insertional mutagenesis with integrating vectors, and saturation of the endogenous miRNA pathway at high shRNA expression levels. Before antiviral RNAi therapies may reach their full clinical potential, the field as a whole has to further optimize delivery vehicles, especially for respiratory targeting.

### 10.3 Antiviral peptides and peptidomimetics

Antiviral peptides (AVPs) fill a unique niche in the antiviral drug discovery landscape by targeting protein-protein interaction surfaces that are resistant to standard small-molecule inhibition. AVPs have a wide range of mechanisms: they can intercalate into and destroy viral envelopes, prevent the conformational rearrangements of viral fusion machinery, prevent viral attachment to host cell receptors, or obstruct post-entry replication processes. In an authoritative overview of new approaches against influenza virus entry, Vanderlinden and Naesens cataloged peptide-based inhibitors that target the endosomal fusion machinery, the receptor-binding site, and the hemagglutinin (HA) stem domain (Vanderlinden and Naesens, 2014). Because the HA stem region is structurally conserved across influenza subtypes and neutralizing antibodies against this epitope are known to provide broad cross-reactive protection, stem-mimetic peptides seek to pharmacologically replicate this neutralization mechanism (**Figure 8**).

Eckert and Kim provided fundamental mechanistic insight into viral membrane fusion by demonstrating that the heptad-repeat (HR) regions of class I fusion proteins undergo dramatic conformational refolding to drive membrane merger, a process that can be inhibited by peptide analogs that compete for the HR grooves and prevent six-helix bundle formation (Eckert and Kim, 2001). This mechanistic paradigm, which was first developed in relation to HIV gp41, has been directly applied to influenza HA2 and has influenced the logical development of a number of peptidic fusion inhibitors. The crystal structure of arbidol, a small molecule with peptidomimetic properties, in complex with influenza HA was resolved by Kadam and Wilson (2017). This revealed a hydrophobic binding pocket at the HA stem interface that is accessible to peptidic ligands that are rationally designed, and it provides a structural template for next-generation fusion inhibitors.

Defensins, cathelicidins, and magainins of amphibian origin are examples of host-derived antimicrobial and antiviral peptides that have shown direct virucidal efficacy against enveloped respiratory viruses. While some AVPs prevent viral uncoating or nuclear import of the ribonucleoprotein complex, their cationic and amphipathic nature permits membrane disruption (Vanderlinden and Naesens, 2014). Poor oral bioavailability, quick proteolytic breakdown in biological fluids, and occasionally unfavorable hemolytic activity are the main drawbacks of natural peptides as therapeutic candidates. These drawbacks are addressed by peptidomimetics, which provide protease resistance while maintaining or improving target affinity through backbone modifications like as β-peptides, peptoids, D-amino acid substitution, and cyclization techniques. De Clercq and Li highlighted the pipeline possibility for AVPs that take advantage of the structural conservation of influenza surface glycoproteins and recorded the variety of clinically licensed antiviral drugs, pointing out a persisting lack of peptide-based drugs for respiratory RNA viruses (De Clercq and Li, 2016). Some of these limitations may be overcome by formulation advancements such as conjugation with cell-penetrating peptide sequences and encapsulation within protease-protective nanocarriers.

### 10.4 Nanoparticle drug delivery systems for enhanced antiviral efficacy

Nanoparticle-based drug delivery systems have emerged as a transformative platform for antiviral therapeutics, capable of addressing pharmacokinetic limitations, achieving site-directed drug release, overcoming biological barriers, and co-delivering multiple therapeutic agents via synergistic mechanisms. The development of antiviral drugs with nanotechnology is more than just a carrier engineering exercise; properly designed nanoparticles can directly interact with virions to exert intrinsic antiviral activity. By functionalizing gold nanoparticles with flexible thiol-terminated ligands that mimicked heparan sulfate moieties, Cagno et al. elegantly illustrated this principle. This resulted in virions engaging in multivalent, irreversible interactions that prevented host cell attachment while also deforming the viral envelope, a virucidal rather than virostatic mechanism applicable across herpes simplex virus, respiratory syncytial virus, dengue virus, and other clinically relevant pathogens (Cagno et al., 2018).

The COVID-19 pandemic spurred nanoparticle antiviral development on an unprecedented scale, resulting in an amazing body of translational data. The nanotechnology toolkit used against SARS-CoV-2, including lipid nanoparticles, polymeric nanoparticles, dendrimers, inorganic nanostructures, and virus-like particles, was thoroughly examined by Chauhan and the colleagues (Chauhan et al., 2020). They listed how each platform type addresses particular aspects of antiviral delivery, such as mucosal targeting, endosomal escape, and immune evasion. Influenza and other respiratory RNA viruses can immediately benefit from the lessons learned from this remarkable translational experiment. Particularly, lipid nanoparticles have showed the ability to carry nucleic acid payloads, such as siRNA, mRNA, and CRISPR-Cas13 components, to the pulmonary epithelium by inhalation routes and have established an excellent clinical safety record. Additional benefits of polymeric nanoparticles based on PLGA (polylactic-co-glycolic acid) and chitosan include mucoadhesion and prolonged drug release. These characteristics are especially helpful in the upper respiratory tract, where mucociliary clearance poses an important barrier to drug retention (**Figure 8**).

Hajj and Whitehead conducted a systematic evaluation of the landscape of non-viral delivery materials for therapeutic RNA and identified several design principles governing effective endosomal escape and cytosolic RNA delivery, including the critical role of ionizable lipid head group chemistry in mediating pH-responsive membrane fusion within endosomes (Hajj and Whitehead, 2017). To improve nanoparticle tropism for virus-infected cells, surface functionalization techniques such as conjugation of viral receptor-binding domain peptides, monoclonal antibody fragments, or pulmonary surfactant proteins are being used. Nanoparticle drug delivery is positioned as an enabling technology across the antiviral pipeline, capable of boosting the potency of various therapeutic modalities, thanks to the ongoing development of inhalable nanoparticle formulations, which are guided by insights from both preclinical respiratory delivery models and COVID-19 clinical data.

### 10.5 mRNA-based therapeutic vaccines: crossover from SARS-CoV-2 experience

One of the most significant developments in contemporary vaccinology is the approval and widespread use of mRNA-based SARS-CoV-2 vaccines within a year of the viral sequence becoming available. This has permanently changed the course of influenza vaccine development. Decades of gradual advancements created the theoretical and scientific groundwork for mRNA vaccines. In a seminal synthesis of the field, Pardi and colleagues described how the addition of nucleoside modifications, specifically N1-methylpseudouridine in place of uridine, abrogates innate immune sensing via toll-like receptors and significantly increases translational efficiency of mRNA *in vivo*, turning a biochemically fragile molecule into a pharmaceutically tractable drug (Pardi et al., 2018). These changes result in mRNA constructs with significantly enhanced stability and protein expression patterns when paired with optimized 5′ cap analogs and 3′ poly(A) tail engineering.

Baden et al. provided the Phase III clinical validation of LNP-encapsulated mRNA vaccines. They showed that the mRNA-1273 vaccine, which targets the SARS-CoV-2 spike protein, achieved about 94% efficacy against symptomatic COVID-19, proving that mRNA-LNP constructs could produce strong humoral and cellular immune responses in a wide range of human populations (Baden et al., 2021). The speed, flexibility, and manufacturing scalability of mRNA vaccine platforms, which are unmatched by traditional egg-based or recombinant protein vaccine technologies, have immediate strategic implications for influenza, where the annual reformulation cycle and persistent antigenic mismatch are still major limitations of current seasonal vaccines. In preclinical models, Chivukula et al. showed that multivalent mRNA vaccine candidates encoding all four influenza strains recommended by the World Health Organization (two influenza A and two influenza B strains) within a single LNP formulation elicited hemagglutination-inhibition titres comparable to or exceeding those of licensed quadrivalent inactivated influenza vaccines, with particular advantages in cross-reactive immune breadth against variant strains (Chivukula et al., 2021).

The mRNA platform is especially promising for the quick delivery of pandemic influenza vaccinations, going beyond seasonal reformulation. A clinical-grade vaccine candidate may theoretically be produced in a matter of weeks after the identification of a pandemic strain sequence due to the modular design of mRNA vaccine manufacturing, a schedule that traditional influenza vaccine manufacturing cannot match. Furthermore, mRNA vaccines that encode influenza antigens other than the rapidly mutating HA head domain, such as the conserved HA stem, neuraminidase, M2e ectodomain, and nucleoprotein, are being investigated as potential universal influenza vaccine candidates that may provide long-lasting cross-protective immunity. Several candidates for next-generation mRNA-based influenza vaccines are presently undergoing clinical investigation thanks to the infrastructure, manufacturing know-how, and regulatory experience gathered during the COVID-19 vaccine deployment (**Figure 8**).

### 10.6 Targeted protein degradation (PROTACs and DEGRADERs) for influenza targets

One of the most conceptually novel deviations from traditional occupancy-based pharmacology to appear in the last 20 years is targeted protein degradation (TPD). TPD techniques reroute the cell’s own ubiquitin-proteasome system (UPS) to specifically eliminate disease-relevant proteins, as opposed to producing a therapeutic impact by sustaining occupancy at an active site or protein–protein interaction interface. Sakamoto et al. first described the prototype technology, Proteolysis-Targeting Chimeras (PROTACs), demonstrating that bifunctional small molecules that could bind a target protein and an E3 ubiquitin ligase at the same time could direct substrate ubiquitination and subsequent proteasomal degradation in a catalytic rather than stoichiometric manner (Sakamoto et al., 2001). A key pharmacological benefit of this catalytic process is that a single PROTAC molecule can participate in several rounds of target degradation, possibly enabling efficient protein depletion at sub-stoichiometric drug concentrations that lower the likelihood of off-target damage.

Bondeson and Crews positioned PROTACs within the larger paradigm of small-molecule-induced protein degradation, enumerating the mechanistic requirements for productive ternary complex formation and defining the structure-activity relationships that govern degrader potency, selectivity, and target resynthesis rates (Bondeson and Crews, 2017). A particularly important discovery was that PROTACs can destroy proteins that were previously thought to be unbreakable, such as transcription factors and scaffolding proteins with poorly defined enzymatic pockets, by targeting surface-exposed areas rather than catalytic sites. The translational aspects of the platform were further developed by Burslem and Crews, who examined the advancement of PROTAC candidates into clinical trials for endocrine and oncology indications and talked about the pharmacokinetic difficulties brought on by the usually high molecular weight and restricted oral bioavailability of bifunctional degrader molecules (Burslem and Crews, 2020).

Applying TPD techniques to influenza and viral infections in general is a developing field with strong mechanistic rationale. The influenza virus relies on many viral proteins that are required for replication yet physically resistant to conventional small-molecule inhibition. High-priority PROTAC targets include the nucleoprotein (NP), which encapsulates the viral genome and is necessary for RNA synthesis, the NS1 non-structural protein, which acts as a versatile interferon antagonist, and the PB2 subunit of the RNA-dependent RNA polymerase complex, which mediates cap-snatching from host pre-mRNAs. Beyond the initial CRBN (cereblon) and VHL (von Hippel-Lindau) ligases, Schapira and coworkers evaluated the growing arsenal of E3 ligase recruiters that can be integrated into degrader designs, significantly expanding the cell-type and tissue-compartment breadth of TPD methods (Schapira et al., 2019). As PROTACs cause target destruction instead of just blocking them, they naturally resist some drug resistance mechanisms. These mechanisms often come from mutations that lower how well an inhibitor binds. As long as the degrader has enough strength to form a working ternary complex, degradation happens. This feature is especially useful in the case of influenza, where the virus’s error-prone polymerase creates resistance variants often. The degradable proteome is further expanded by DEGRADERs, a word that includes molecular glues, hydrophobic tagging techniques, and LYTAC (lysosome-targeting chimera) technologies for extracellular and membrane protein targets. It may also apply to influenza surface glycoproteins. The combination of structural virology data showing ligandable surfaces on influenza proteins, growing libraries of E3 ligase recruiters, and better computational tools for predicting ternary complexes makes TPD a promising new strategy for discovering drugs for influenza.

### 10.7 Future perspectives

Several interconnected priorities will determine how quickly the evidence gaps identified throughout this review can be closed: regulatory pathways for emerging platforms, adaptive trial infrastructure, and the design of broad-spectrum, resistance-resilient antiviral agents.

The major translational challenge across all three platforms (presented in this section) is regulatory precedent in the context of an acute infectious disease indication. RNAi therapeutics have an approved pathway, with all of the approved siRNA products so far being used to treat chronic genetic diseases delivered intravenously or subcutaneously, respectively, but there is no inhaled or pulmonary RNAi product that has ever been approved. It means that the most relevant delivery system for influenza, either inhaled or pulmonary, is without precedent. In May 2026, PROTACs made a real breakthrough with the first FDA approval of a protein degrader drug, vepdegestrant, to treat ESR1-mutated breast cancer (Arvinas announces FDA approval of VEPPANU (vepdegestrant) for the treatment of ESR1m, ER+/HER2- advanced breast cancer | Arvinas). But none of these precedents directly applies to the safety and chemistry-manufacturing controls that an inhaled, short-course PROTAC for an acute, self-limiting respiratory infection in an otherwise healthy population would have to meet in order to be approved. The three most promising regulatory strategies are for existing FDA frameworks to be adapted to *in vivo* or ex vivo permanent genome editing for rare monogenic diseases, or for a new ‘plausible mechanism’ pathway to be developed for bespoke gene editing therapies; both forms of editing were designed for rare monogenic diseases, where the standard of evidence is granted under the 2023 orphan-disease approval framework (U.S. Food and Drug Administration, 2024; Prasad and Makary, 2025). None of these precedents neatly fit the case of an acutely delivered RNA-guided nuclease to treat a fairly common, self-limited infection in a large, general population. In this regard, an antiviral CRISPR-Cas13 would need an FDA review division and a documentary process that is not yet in place. Addressing this regulatory shortcoming, in addition to delivery and manufacturing challenges mentioned above, will be as critical as continued mechanistic optimization is for clinical translation of these platforms.

The lack of evidence documented across all sections of this review, especially in the case of critically ill and special populations (Special Populations section), is largely due to the fact that traditional trial designs cannot recruit significant numbers of participants in a single, unpredictable influenza season. Adaptive platform trials are not a quick acceleration of a similar approach, but a structure. The Influenza Antiviral Domain of REMAP-CAP, originally developed for community-acquired pneumonia, has been an active arm of REMAP-CAP that has randomized patients with confirmed influenza to various antiviral therapies at the same time, while they were receiving routine care for other pneumonia-related illnesses in the ICU, in over 20 countries (Angus et al., 2020). This is an embedded, always on design, just the sort of infrastructure this review has identified over and over again as missing: a mechanism that can generate randomized evidence in populations that have now been systematically excluded from the standard registration trials that inform current antiviral guidance, such as the critically ill, immunocompromised, and patients on organ support.

Instead of screening a single type of neuraminidase, a computational pipeline was used to search almost 500,000 compounds against the neuraminidases of all of the influenza A group 1, group 2 and influenza B types, and a cross-subtype molecular dynamics simulation was used to validate potential hits. This strategy led to the discovery of 10 compounds that bound to each of the three neuraminidase backbones including a diastereomer of zanamivir that was “discovered” independently from the screen. Importantly, the same candidates were tested with the H275Y and D151G resistance mutations, and these candidates were found to have stable binding while the oseltamivir binding mode shifted from the hydrophobic contacts that are altered by these mutations to an extensive hydrogen-bond network with the conserved electrostatic core of the binding site (Gevorgyan et al., 2026). This helps to make more concrete the idea of a resistance-resilient target: a binding mode “anchored” to residues which the virus is not capable of changing without losing its catalytic activity, rather than to peripheral contacts which can be easily modified by only a single change. What has been shown is so far not influenza-specific but rather against conserved structural pockets across different families of RNA viruses, such as hepatitis C virus and human coronavirus 229E, highlighting the potential for this resistance-resilient design approach to be extended to other conserved targets in viruses, not just NA-targeted small molecules (Umansky et al., 2025).

## 11 Conclusion

The history of the development of antiviral drugs against influenza is characterized by the consistent pharmacological advancement due to the extraordinary adaptability of the virus. As adamantanes became largely obsolete with widespread resistance to the ongoing use of neuraminidase inhibitors (NAIs) and more recently with the advent of polymerase-directed drugs like baloxavir marboxil, every new treatment has increased clinical opportunities as well as exposed the enduring limitations in efficacy, resistance, and evidence.

NAIs are still clinically useful especially in high-risk groups and when used early in infection, although their minimal therapeutic effect in otherwise healthy individuals and time dependence limits their overall effect. Despite the fact that global susceptibility has remained mostly constant, the experience of the past with resistant strains demonstrates that it is necessary to remain vigilant. Baloxavir has significant benefits, such as single-dose regimen and high efficacy in reducing viral load. But resistance to treatment and low efficacy in severe disease highlights the importance of optimized strategies in its use. The recent regulatory approval of suraxavir marboxil and ZX-7101A in China as next-generation CENIs, along with onradivir as the first approved PB2 inhibitor, shows that the PA and PB2 endonuclease targets are still clinical useful. It also shows that optimizing existing drug classes at the scaffold level can lead to drugs with better resistant profiles, which expands the polymerase inhibitor arsenal beyond baloxavir monotherapy. Combination therapy is a potential strategy to improve antiviral activity and raise the genetic barrier to resistance though it needs to be strongly clinically validated.

Host-directed therapies represent an alternative approach that is interesting as it targets conserved cellular pathways and may reduce the potential resistance risk, alongside direct-acting antivirals. Nonetheless, their clinical translation is at its initial stages and requires dedicated clinical evaluation. Meanwhile, the ever-present risk of highly pathogenic strains of avian influenza indicates severe flaws in current preparedness, such as the absence of high-quality clinical data and excessive dependence on a small number of antivirals. Among repurposed agents, diltiazem in combination with baloxavir and NTZ in combination with oseltamivir represent the most clinically actionable near-term combinations, requiring only adaptive trial validation rather than new drug development.

The H5N1 pandemic threat combines all these shortcomings: a therapy toolbox based on a single agent whose effectiveness remains largely unproved, a resistance problem that makes single-agent therapy ineffective, biologics pipelines blocked by mucosal delivery problems, and a new and exciting platforms landscape that provides mechanistic innovation but has not yet been tested in a clinical setting. Pandemic preparedness in 2025 is not a pharmacological problem; the scientific tools exist. It is a translational one. The adaptive trial infrastructure, equitable stockpiling architecture, and real-time pharmacovigilance networks needed to deploy those tools in a pandemic’s first weeks remain underdeveloped. The challenge for the next five years in influenza antiviral research is to fill this gap.

New modalities like RNA-based therapies and CRISPR-associated systems are promising in the long term but have significant translational issues. Together, these trends suggest a future model of antiviral care based on combination approaches, greater antiviral spectrum, and flexible therapeutic platforms. This vision will be achieved through a long-term investment in research, enhanced clinical trial facilities, and fair global access to antivirals to have a more effective and coordinated response to future influenza threats.
